# Mapping Immune-Inflammatory Niches on Zirconia Bone Implants: Single-Cell Transcriptomic Profiling

**DOI:** 10.34133/research.1162

**Published:** 2026-03-10

**Authors:** Jiannan Zhou, An Li, Jiahao Chen, Jingtao Dai, Wentai Zhang, Zhilu Yang, Ping Li

**Affiliations:** ^1^ School and Hospital of Stomatology, Guangdong Engineering Research Center of Oral Restoration and Reconstruction & Guangzhou Key Laboratory of Basic and Applied Research of Oral Regenerative Medicine, Guangzhou Medical University, Guangzhou, China.; ^2^ Department of Periodontology, Stomatological Hospital, School of Stomatology, Southern Medical University, Guangzhou, China.; ^3^ Department of Prosthodontics, Geriatric Dentistry and Craniomandibular Disorders, Charité-Universitätsmedizin Berlin, Corporate Member of Freie Universität Berlin and Humboldt-Universität zu Berlin, Berlin, Germany.; ^4^ Department of Orthodontics, Stomatological Hospital, School of Stomatology, Southern Medical University, Guangzhou, China.; ^5^ The Tenth Affiliated Hospital, Southern Medical University (Dongguan People’s Hospital), Dongguan, Guangdong 523059, China.

## Abstract

Zirconia (ZrO_2_) has become a promising alternative to titanium (Ti) for bone implants due to its excellent biocompatibility. Despite this, the osseointegration of ZrO_2_ remains lower than that of Ti implants. However, the underlying biological mechanisms, particularly the osteoimmune response, remain not fully elucidated. Herein, we employed single-cell RNA sequencing to profile the immune-inflammatory niches of ZrO_2_ and Ti-based implants, to elucidate mechanisms that could guide the osteogenic functionalization of ZrO_2_ implants. The analysis provides a high-resolution atlas of immune–stromal cell dynamics at the bone–implant interface, identifying distinct cellular subsets and ligand–receptor axes activated by each material. Ti implants preferentially enriched stem-cell niches and up-regulated collagen organization through fibroblast-specific collagen type I alpha 1 chain/syndecan 1 signaling, promoting regenerative extracellular matrix remodeling and early osteogenic microenvironment. In contrast, ZrO_2_ implants triggered lymphoid-dominated responses, characterized by collagen type VI alpha 2 chain/cluster of differentiation 44-mediated macrophage activation, and pro-inflammatory pathway activation. In vivo validation via bulk RNA sequencing confirmed these material-specific immunomodulatory programs, with Ti favoring osteogenic microenvironments and ZrO_2_ inducing fibro-inflammatory niches. These findings provide mechanistic targets for designing immunomodulatory biointerfaces to enhance the osseointegration of ZrO_2_ implants.

## Introduction

Zirconia (ZrO_2_) has garnered substantial attention in the field of oral implantology as a promising alternative to metallic titanium (Ti) implants [1]. This interest stems from its superior aesthetic properties, biocompatibility, and potential to reduce metal ion release, which are desirable factors for long-term implant success and patient satisfaction. However, despite these advantages, clinical and preclinical studies have consistently demonstrated that ZrO_2_ implants exhibit slower osseointegration in comparison with Ti counterparts [2,3]. Osseointegration is characterized by the formation of a stable bond between bone and implant, where bone tissue grows directly onto the implant surface without the involvement of fibrous tissue, which is a pivotal determinant of implant stability and longevity [4–8].

The difference in osseointegration among materials may arise from variations in surface topography, hydrophilicity, and protein adsorption patterns [9]. These factors are thought to affect the initial attachment, proliferation, and differentiation of osteoblasts, ultimately resulting in the formation of a mineralized bone matrix around the implant [8]. More importantly, material-specific immune responses play a vital yet incompletely understood role in tissue regeneration and osseointegration [10,11]. Emerging evidence suggests that initial immune activation not only triggers the inflammatory cascade but also critically regulates cellular recruitment, differentiation trajectories, and extracellular matrix (ECM) remodeling, all essential for successful osteogenesis and vascularization [12]. Although considerable progress has been made in elucidating material-specific immune responses in osseointegration, the complex interaction between implant materials and the initial immune-regulated processes of tissue regeneration remains largely unexplored.

The advent of single-cell RNA sequencing (scRNA-seq) has revolutionized our ability to dissect complex biological processes with high resolution [13,14]. In the osteoimmune microenvironment, scRNA-seq enables detailed characterization of the heterogeneity and dynamics of immune and stromal cell populations involved in bone regeneration and osseointegration [15]. Its application to material–cell interactions has uncovered key cellular subsets and molecular pathways governing implant–tissue cross talk [16,17]. Li et al. [17] employed scRNA-seq to investigate the osteoimmune microenvironment following biomaterial implantation in mice, revealing strong cellular communication between neutrophils and hematopoietic stem cells via the C-X-C motif chemokine ligand 12/C-X-C motif chemokine receptor 3 axis, providing design insights for improving osseointegration. However, the osteoimmune microenvironment associated with ZrO_2_ implant osseointegration—particularly the material-specific immune–stromal cross talk networks—and its regulatory mechanisms on osseointegration remain inadequately characterized.

**Fig. 1.**
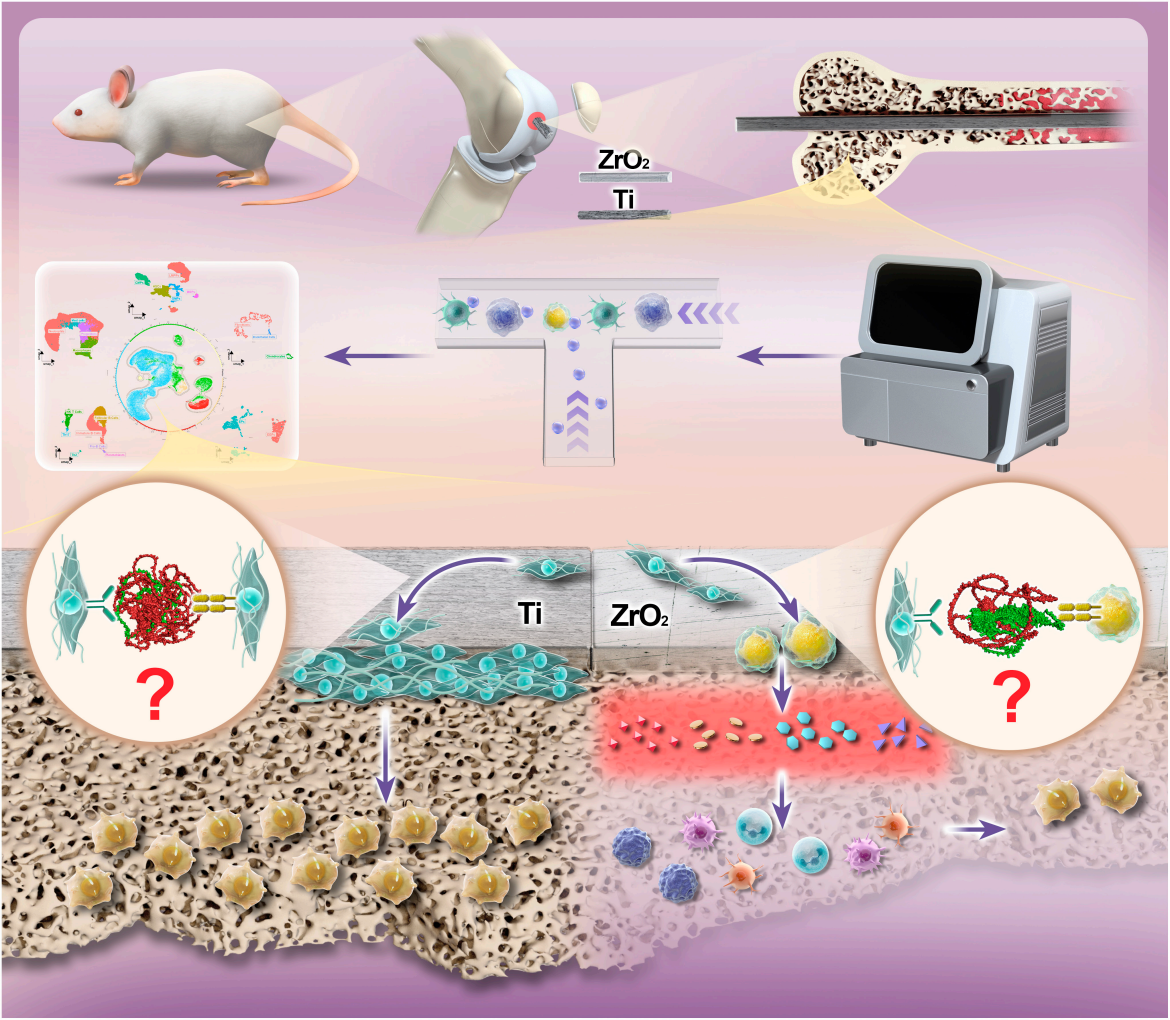
Divergent immune and osteogenic responses to titanium (Ti) versus zirconia (ZrO_2_) implants in the bone microenvironment, determined by single-cell transcriptomics.

To address these gaps, this study elucidates the mechanisms underlying the differential osseointegration capabilities of Ti and ZrO_2_ implant materials within the framework of cell–material interactions, aiming to guide the osteogenic functionalization of ZrO_2_ implants. By focusing on the active cells present on implant surfaces, this study examined how the intrinsic properties of Ti and ZrO_2_ regulate cellular behavior in the early osteoimmune microenvironment. To this end, the scRNA-seq technology was employed to construct a detailed single-cell atlas of the bone-marrow microenvironment early postimplantation of each of these implants, with a focus on identifying key cellular subsets, gene expression patterns, and signaling pathways that are differentially regulated (Fig. 1). Specific ligand–receptor pairs were further validated in the bone-marrow microenvironment via bulk RNA sequencing (bulk RNA-seq). Our findings provide mechanistic targets for the rational design of immunomodulatory biointerfaces to enhance the osseointegration of ZrO_2_ implants.

## Results

### Surface characteristics and biocompatibility of the Ti and ZrO_2_ implants

**Fig. 2.**
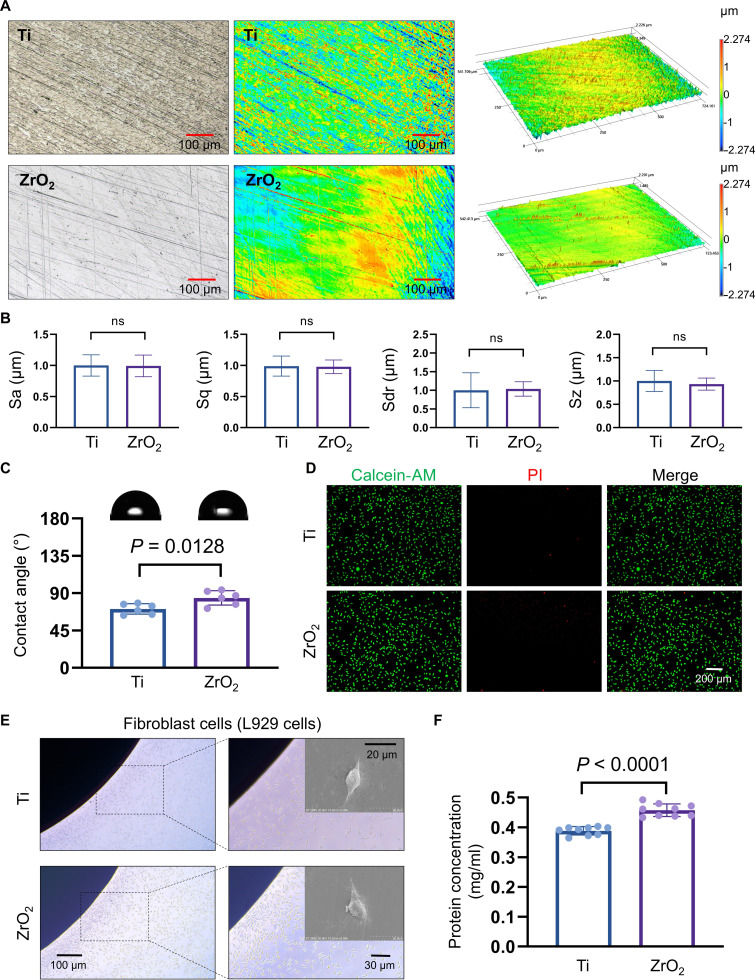
Characterization of the titanium (Ti) and zirconia (ZrO_2_) materials. (A and B) Surface roughness and corresponding quantitative analysis, respectively. Sa (arithmetic mean height), Sq (root mean square height), Sdr (developed interfacial area ratio), and Sz (maximum height). Scale bars: 100 μm. (C) Contact-angle measurement and quantitative analysis of the material surfaces by using the pendant-drop method. (D) Viability assessment of L929 cells cultured with 72-h material extracts using calcein-acetoxymethyl ester (calcein-AM)/propidium iodide (PI) staining. Scale bars: 200 μm. (E) Cell-adhesion abilities of the material surfaces, showing direct cell contact. Scale bars: 100, 30, and 20 μm. (F) Adherent-protein concentrations on the material surfaces. Statistical significance was assessed using the *t* test. ns, not significant.

The surface roughness of each sample was recorded using a noncontact confocal laser scanning microscope at 20× magnification, with the following calculated parameters: Sa (arithmetic mean height), Sq (root mean square height), Sdr (developed interfacial area ratio), and Sz (maximum height) (Fig. 2A and B). Statistical analysis revealed no significant differences in Sa, Sq, Sdr, or Sz between the Ti and ZrO_2_ surfaces (*P* > 0.05). Field emission scanning electron microscope (SEM) characterization revealed that both Ti and ZrO_2_ surfaces exhibited textured microstructures (Fig. S1A). The hydrophilicity of Ti and ZrO_2_ was assessed using the pendant-drop method. The contact angle of the ZrO_2_ surface (84.2° ± 1.1°) was significantly higher than that of the Ti-based surface (70.9° ± 1.2°), indicating comparatively lower surface wettability (Fig. 2C).

Energy-dispersive x-ray spectroscopy elemental mapping confirmed the homogeneous distributions of the primary constituents. The primary constituent of the Ti surfaces was titanium, which was accompanied by minor amounts of V, C, O, and Al. In contrast, elemental mapping of the ZrO_2_ surfaces revealed a homogeneous distribution of Zr, O, and C (Fig. S1B to F).

In vitro biological assessments were conducted using L929 fibroblast cells. After a 72-h incubation in Ti and ZrO_2_ extracts, minimal cell death was observed in viability assays, suggesting no obvious cytotoxicity (Fig. 2D). Cell-adhesion experiments revealed extensive cellular attachment and spreading on both Ti and ZrO_2_ substrates, confirming favorable cytocompatibility (Fig. 2E). The protein adsorption tests further demonstrated that the adsorbed albumin concentration on ZrO_2_ implant surfaces (~0.46 mg/ml) was significantly higher compared to that on Ti surfaces (~0.39 mg/ml), suggesting enhanced protein adsorption capability of ZrO_2_ implant materials (Fig. 2F).

### Early postimplantation cellular landscapes of the bone marrow surrounding the Ti and ZrO_2_ implants

**Fig. 3.**
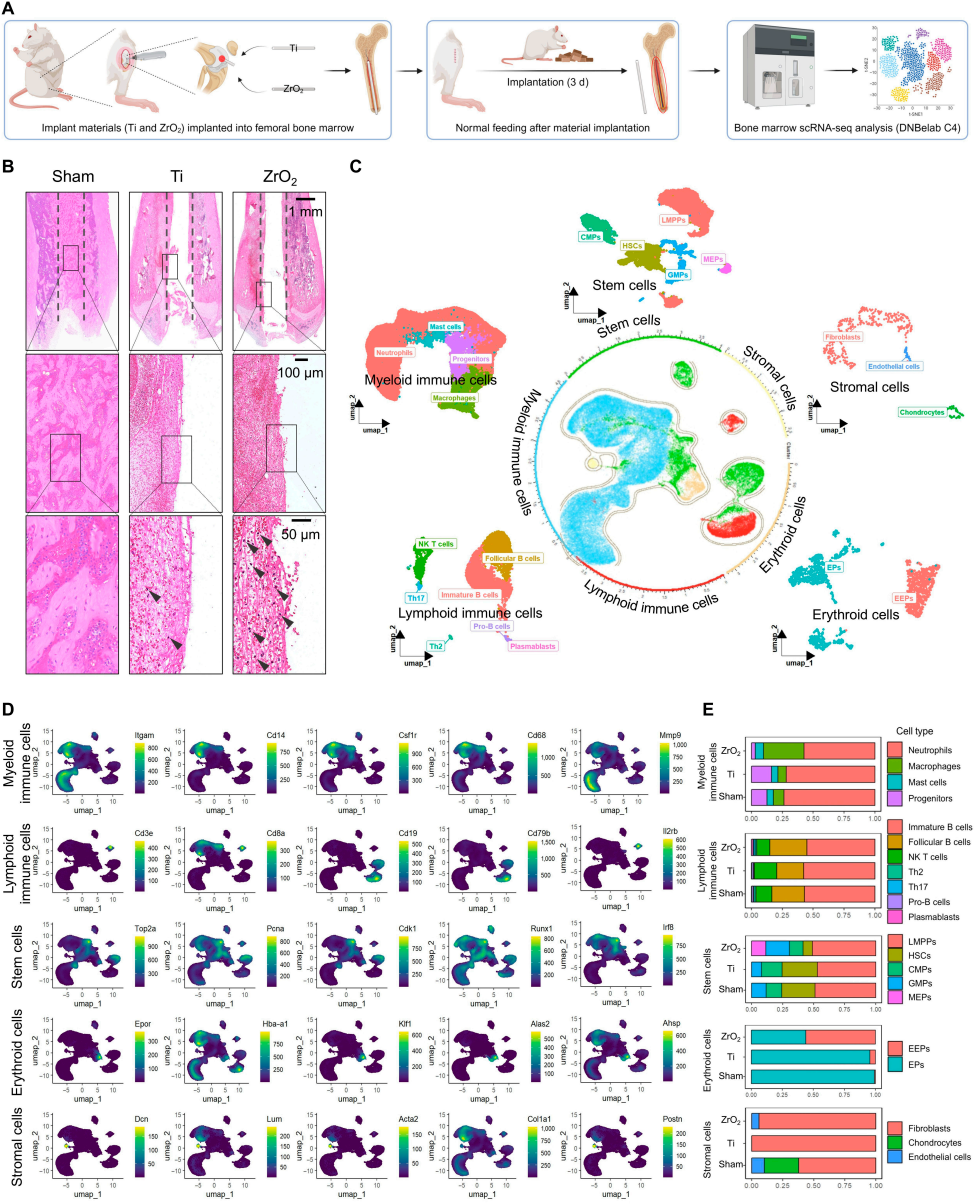
Histological analyses and cellular atlases of the bone marrow surrounding the titanium (Ti) and zirconia (ZrO_2_) implants. (A) Schematic diagram of the rat model of intramedullary femoral implantation and the experimental workflow. (B) Hematoxylin-and-eosin staining of bone marrow. Dashed line: implant material contour. Arrow: inflammatory cells. Scale bars: 1 mm, 100 μm, and 50 μm. (C) Bone-marrow cell atlas and their subpopulations. (D) Spatial expression of classical cell-marker genes. (E) Proportional distributions of bone-marrow cells and their subpopulations across the sham, Ti, and ZrO_2_ groups. scRNA-seq, single-cell RNA sequencing; NK, natural killer; HSCs, hematopoietic stem cells; LMPPs, lympho-myeloid primed progenitors; CMPs, common myeloid progenitors; GMPs, granulocyte-monocyte progenitors; MEPs, megakaryocyte–erythroid progenitors; EPs, erythroid progenitors; EEPs, early erythroid progenitors.

To investigate the early cellular changes in the bone-marrow microenvironment following Ti and ZrO_2_ implantation, a rat model of intramedullary femoral implantation was established. The bone marrow surrounding the implants was collected 3 d postimplantation for scRNA-seq and histological analysis (Fig. 3A and Fig. S2A and B). Hematoxylin-and-eosin (H&E) staining of the bone marrow revealed the presence of fibroblasts and collagen-fiber formation in both the Ti- and ZrO_2_-implanted groups (Fig. 3B). Notably, fewer immune cells were detected around the Ti implants than around the ZrO_2_ implants.

Following preprocessing of the scRNA-seq data, multiplets and low-quality cells were removed, yielding a total of 66,159 cells from the bone marrow surrounding the implants (Fig. S2C). Principal component analysis and Harmony batch-effect correction identified 19 distinct cell clusters (Fig. S2D to F). Based on differentially expressed genes (DEGs) and known markers, a comprehensive cell atlas and subpopulation map were constructed using the following marker genes: for myeloid cells, *Itgam*, *Cd14*, *Csf1r*, *Cd68*, and *Mmp9*; for lymphoid cells, *Cd3e*, *Cd8a*, *Cd19*, *Cd79b*, and *Il2rb*; for stem cells, *Top2a*, *Pcna*, *Cdk1*, *Runx1*, and *Irf8*; for erythroid cells, *Epor*, *Hba-a1*, *Klf1*, *Alas2*, and *Ahsp*; and for stromal cells, *Dcn*, *Lum*, *Acta2*, *Col1a1*, and *Postn* (Fig. 3C and D and Fig. S2G and H).

Cell type proportions were visualized in the sham, Ti, and ZrO_2_ groups to assess the cellular changes around the implants (Fig. 3E). The results showed that cell type proportions were largely similar between the sham and Ti groups, with only minor variations, such as fewer lymphoid cells and more stem cells in the Ti group than in the sham group. In contrast, the ZrO_2_ group exhibited marked changes, with increased proportions of lymphoid and erythroid cells, alongside a marked reduction in stem cells, compared with those in the sham group. These findings suggest that Ti induces a bone-marrow microenvironment response similar to that of the sham group, whereas ZrO_2_ elicits a distinct cellular reaction.

### Fibroblastic COL1A1/SDC1 signaling mediates the Ti-implant-induced regenerative responses

**Fig. 4.**
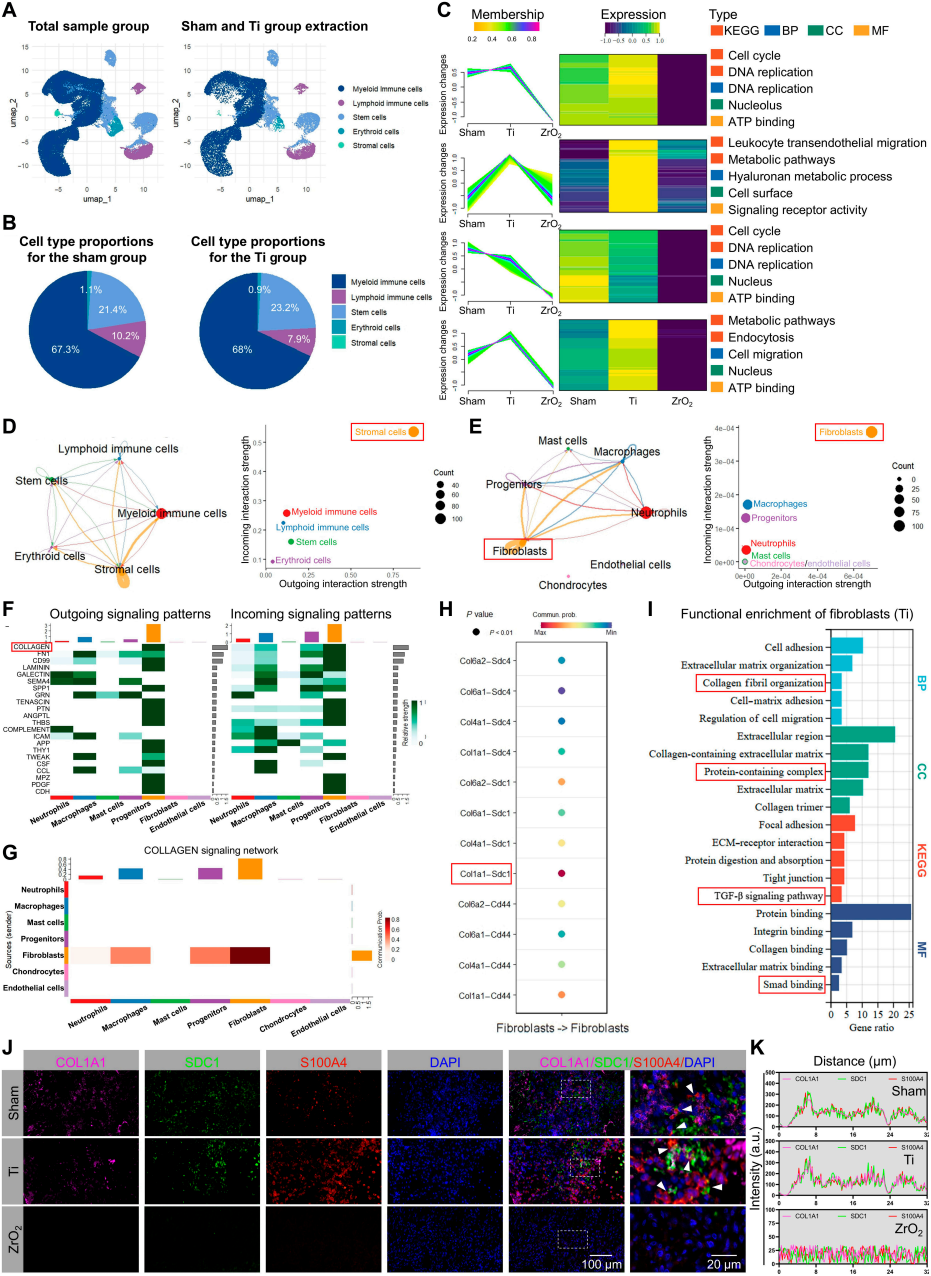
Intercellular cross talk and functional enrichment in the bone-marrow microenvironment following titanium (Ti) implantation. (A and B) Cells extracted from the Ti implant (A) and their proportions compared with those in the sham group (B). (C) Fuzzy *C*-means clustering of the differentially expressed genes (DEGs) and their functional annotation via Kyoto Encyclopedia of Genes and Genomes (KEGG) and Gene Ontology (GO) enrichment analyses. BP, Biological Process; CC, Cellular Component; MF, Molecular Function. (D and E) Outgoing–incoming cross talk analysis among the bone-marrow cells and their subpopulations. (F) Outgoing–incoming signaling patterns and associated interaction strengths. (G) Analysis of collagen-related signaling networks and intercellular interaction strengths. (H) Interaction-strength analysis of candidate ligand–receptor pairs in bone-marrow fibroblasts. (I) KEGG and GO (BP, CC, and MF) enrichment analyses of the DEGs in bone-marrow fibroblasts. (J and K) Multiplex fluorescence colocalization and costaining analysis of candidate ligand–receptor pairs. Scale bars: 100 and 20 μm. COL1A1, collagen type I alpha 1 chain; SDC1, syndecan 1; ATP, adenosine triphosphate; DAPI, 4′,6-diamidino-2-phenylindole.

To reveal the specific cellular and molecular programs induced by each implant material, we assessed the bone-marrow cellular composition surrounding the respective implants across groups. The results showed a decrease in lymphocyte number and an increase in stem-cell number in the Ti group, indicating a strong early proliferative response in the bone-marrow microenvironment following Ti implantation (Fig. 4A and B). The DEGs (compared with the ZrO_2_ group) between the 2 groups were analyzed using fuzzy *C*-means clustering, revealing that gene clusters were predominantly enriched in the Ti group (Fig. 4C). Subsequent Kyoto Encyclopedia of Genes and Genomes (KEGG) and Gene Ontology (GO) enrichment analyses of these clustered genes showed significant enrichment in pathways related to the cell cycle, DNA replication, adenosine triphosphate (ATP) binding, metabolic pathways, hyaluronan metabolism, and cell migration (*P* < 0.01). These findings indicate that, in the early postimplantation stage, Ti promotes cellular proliferation, active energy metabolism, and dynamic ECM remodeling within the bone-marrow microenvironment.

To examine intercellular cross talk around Ti implants, cell–cell interactions were analyzed. The results demonstrated that stromal cells displayed comparatively elevated levels of both outgoing and incoming intercellular interactions (Fig. 4D and Fig. S3A to E). Among stromal cells, fibroblasts demonstrated the strongest cross talk capabilities (Fig. 4E), suggesting that fibroblasts may exert functional roles through autocrine signaling after Ti implantation. Similarly, collagen-related signaling pathways played a dominant role in the Ti group, further supporting fibroblast–fibroblast interactions (Fig. 4F and G). Notably, the specific ligand–receptor pair collagen type I alpha 1 chain/syndecan 1 (COL1A1/SDC1) was identified as the strongest interaction pair in fibroblasts (Fig. 4H). To further explore fibroblast function around Ti implants, DEGs (compared with the expression levels of all cells from other groups) in fibroblasts were extracted separately for KEGG and GO enrichment analyses. The results revealed significant enrichment in pathways associated with dynamic ECM remodeling, coordinated regulation of cell migration and adhesion, and the anti-inflammatory role of the transforming growth factor beta (TGF-β) signaling pathway (*P* < 0.05) (Fig. 4I). These findings align with those from the fuzzy *C*-means clustering analysis, suggesting that in the early postimplantation stage, Ti implants support a regenerative bone-marrow microenvironment.

Molecular dynamics simulations and docking analyses of COL1A1 and SDC1 by using PyMOL revealed multiple high-affinity binding sites, indicating strong interaction potential (Fig. S3F). Multiplex immunofluorescence staining further confirmed the colocalization of COL1A1 and SDC1 in the fibroblasts (S100A4^+^) surrounding the Ti implant. In contrast, only partial colocalization was observed in the sham group, and no such interaction was detected in the ZrO_2_ group (Fig. 4J and K). These findings suggest that following Ti implantation, bone-marrow fibroblasts secrete COL1A1, which binds to its surface receptor SDC1, potentially activating the TGF-β signaling pathway.

### ZrO_2_ implants orchestrate a profibrotic inflammatory niche via fibroblast–macrophage cross talk mediated by the COL6A2/CD44 axis

**Fig. 5.**
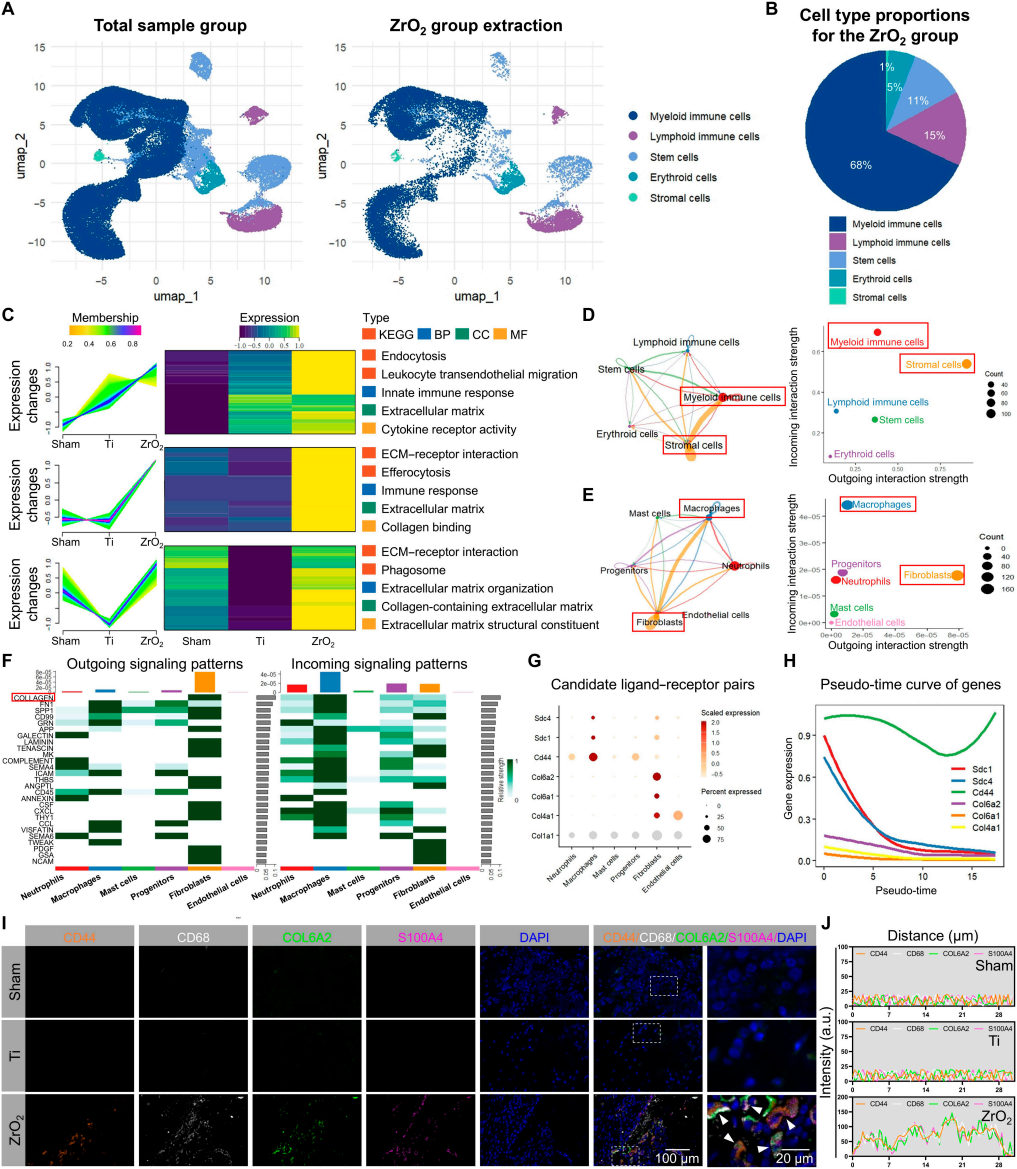
Intercellular cross talk in the bone-marrow microenvironment following zirconia (ZrO_2_) implantation. (A and B) Cells extracted from the ZrO_2_ implant (A) and their proportions (B). (C) Fuzzy *C*-means clustering of the DEGs in bone-marrow cells between the ZrO_2_ groups, and their functional annotation via KEGG and GO enrichment analyses. (D and E) Outgoing–incoming cross talk analysis among the bone-marrow cells and their subpopulations. (F) Outgoing–incoming signaling patterns and associated interaction strengths. (G) Analysis of candidate ligand–receptor pairs among the cells. (H) Temporal expression patterns of candidate ligand–receptor pairs. (I and J) Multiplex fluorescence colocalization and costaining analysis of ligand–receptor pairs. Scale bars: 100 and 20 μm. COL6A2, collagen type VI alpha 2 chain.

Given the observed distinct cellular responses between the implant materials, the cellular composition of the bone marrow surrounding the ZrO_2_ implants was therefore characterized (Fig. 5A and B). To assess the impact of ZrO_2_ implantation on the bone-marrow microenvironment, the DEGs (compared with the Ti group) in the extracted cells were subjected to fuzzy *C*-means clustering, followed by KEGG and GO enrichment analyses (Fig. 5C). The results revealed significant enrichment in pathways associated with the innate immune response, leukocyte transendothelial migration, immune activation, ECM–receptor interactions, collagen binding, collagen-containing ECM, and ECM organization (*P* < 0.01), collectively indicating potential fibrotic and inflammatory responses in the ZrO_2_ group. Cell–cell interaction analysis revealed robust cross talk between stromal and myeloid cells, with stromal cells exhibiting the highest outgoing interaction strength and myeloid cells receiving the strongest incoming signals (Fig. 5D and Fig. S4A to E). Further dissection of these interactions identified fibroblasts as the predominant signal-sending population and macrophages as the principal recipients, suggesting fibroblast-to-macrophage signaling following ZrO_2_ implantation (Fig. 5E).

Cross talk analysis between fibroblasts and macrophages identified collagen as the dominant signaling pathway (Fig. 5F and Fig. S4F). Within this pathway, collagen type VI alpha 2 chain (COL6A2) was the most highly expressed ligand in fibroblasts, whereas CD44 was the most abundantly expressed receptor in macrophages (Fig. 5G). Results from gene-trajectory analysis showed consistently high expression trends of COL6A2 and CD44 (Fig. 5H). To validate this ligand–receptor interaction, AlphaFold3 was employed to predict the COL6A2 protein structure, and subsequent molecular dynamics simulations and docking analyses using PyMOL suggested multiple high-affinity binding sites between COL6A2 and CD44 (Fig. S4G to I). Multiplex immunofluorescence staining confirmed the colocalization of COL6A2 on fibroblasts (S100A4^+^) and CD44 on macrophages (CD68^+^) in the ZrO_2_ group, a pattern not observed in the sham or Ti group (Fig. 5I and J).

To functionally validate the regulatory role of fibroblasts via the COL6A2–CD44 axis in macrophage activation and the formation of a pro-inflammatory microenvironment, we assessed the expression of nitric oxide synthase 2 (NOS2) (inducible nitric oxide synthase), a canonical marker for inflammatory macrophages. Western blot analysis revealed a significant up-regulation of NOS2 protein in the ZrO_2_ group compared to those in the Ti and sham groups, where its expression remained unchanged (Fig. S5A and B). Consistently, quantitative real-time polymerase chain reaction analysis demonstrated a congruent up-regulation at the transcriptional level (Fig. S5C). Furthermore, immunofluorescence staining confirmed the pronounced localization of inflammatory macrophages in the peri-implant bone marrow of the ZrO_2_ group, which was markedly less evident in the sham and Ti groups (Fig. S5D and E). These results further substantiate that ZrO_2_ implantation, potentially via the fibroblast–macrophage COL6A2–CD44 communication axis, drives the polarization of macrophages toward a pro-inflammatory phenotype, thereby establishing an immune microenvironment conducive to inflammation rather than osseointegration.

### Bulk RNA-seq indicates differential signaling in Ti and ZrO_2_ implants

**Fig. 6.**
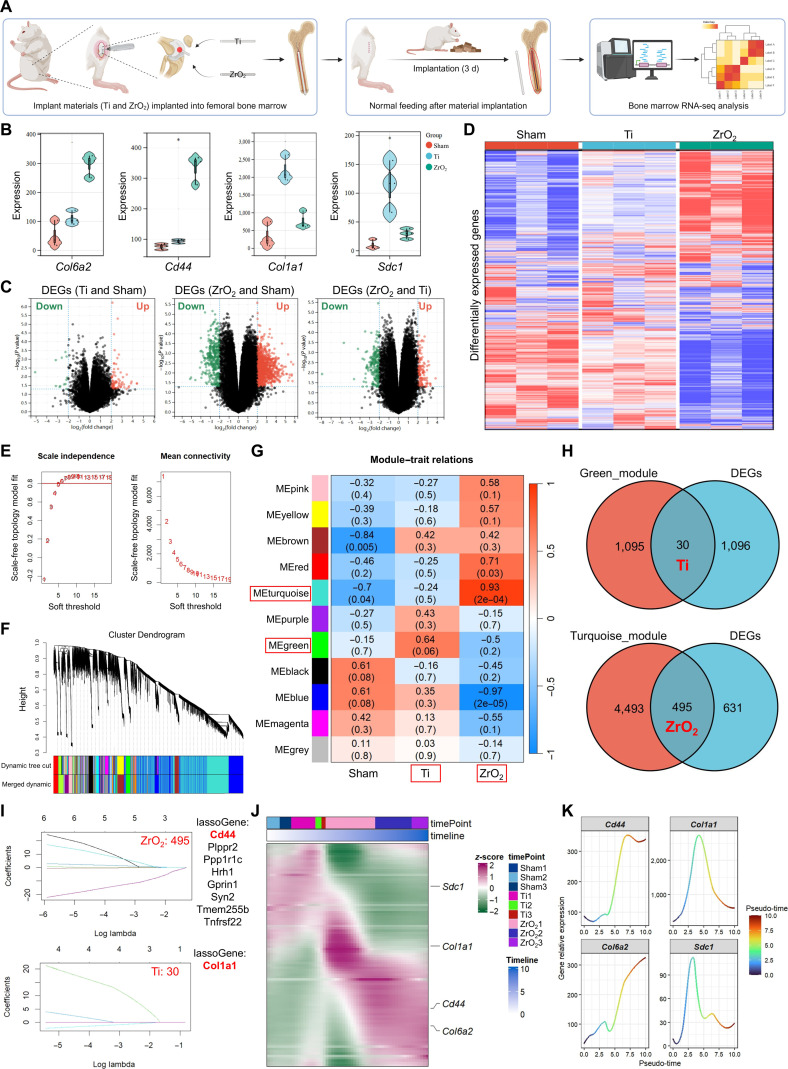
Validation of the specific ligand–receptor pairs between the implants and bone-marrow cells. (A) Schematic diagram of the rat model of intramedullary femoral implantation and bulk RNA sequencing workflow. (B) Violin plots showing the expression levels of *Col6a2*, *Cd44*, *Col1a1*, and *Sdc1*. (C and D) Hierarchical clustering heatmaps of pairwise DEGs among the sham, Ti, and ZrO_2_ groups. (E to G) Identification of the gene modules associated with Ti and ZrO_2_ via weighted gene coexpression network analysis. (H) Overlapping genes between module-related genes and DEGs in each group. (I) Contributions of Ti and ZrO_2_-associated feature genes to predictive outcomes, assessed using least absolute shrinkage and selection operator (LASSO) regression analysis. (J and K) Temporal expression patterns and correlations of *Col6a2*, *Cd44*, *Col1a1*, and *Sdc1*. Statistical significance was assessed using the *t* test.

Based on the above findings, a rat model of intramedullary femoral implantation for Ti and ZrO_2_ was established, followed by bulk RNA-seq analysis to validate the ligand–receptor associations identified via scRNA-seq (Fig. 6A). Expression analysis of the ligand–receptor pairs in the Ti and ZrO_2_ groups revealed that *Col6a2* and *Cd44* were significantly up-regulated in the ZrO_2_ group, whereas *Col1a1* and *Sdc1* were significantly up-regulated in the Ti group, consistent with previous results (Fig. 6B). Pairwise comparisons between the sham, Ti, and ZrO_2_ groups were performed to identify DEGs, which were then visualized using hierarchical clustering analysis (Fig. 6C and D). The results showed similar clustering patterns between the sham and Ti groups, whereas the ZrO_2_ group exhibited a distinct profile, suggesting divergent impacts of Ti and ZrO_2_ implants on the bone-marrow microenvironment. To further investigate the associations between COL6A2/CD44 and COL1A1/SDC1 in the Ti and ZrO_2_ groups, weighted gene coexpression network analysis (WGCNA) was performed to identify gene modules (Fig. 6E and F). The green module showed a correlation coefficient of 0.64 with the Ti group, whereas the turquoise module showed a strong correlation of 0.93 with the ZrO_2_ group, indicating robust associations between these modules and their respective implant types (Fig. 6G).

Subsequently, 495 shared genes were identified between the turquoise module and the DEGs of the ZrO_2_ group, whereas 30 shared genes were identified between the green module and the DEGs of the Ti group (Fig. 6H). Feature genes were further evaluated using least absolute shrinkage and selection operator (LASSO) regression analysis, which revealed that *Cd44* was the most significant contributor in the ZrO_2_ group, whereas *Col1a1* was the key contributor in the Ti group (Fig. 6I). Finally, temporal gene expression analysis showed consistent expression trends between *Col6a2* and *Cd44* in the ZrO_2_ group and between *Col1a1* and *Sdc1* in the Ti group, further supporting our scRNA-seq findings (Fig. 6J and K).

### Ti and ZrO_2_ implants differentially regulate early bone healing and immune inflammation

**Fig. 7.**
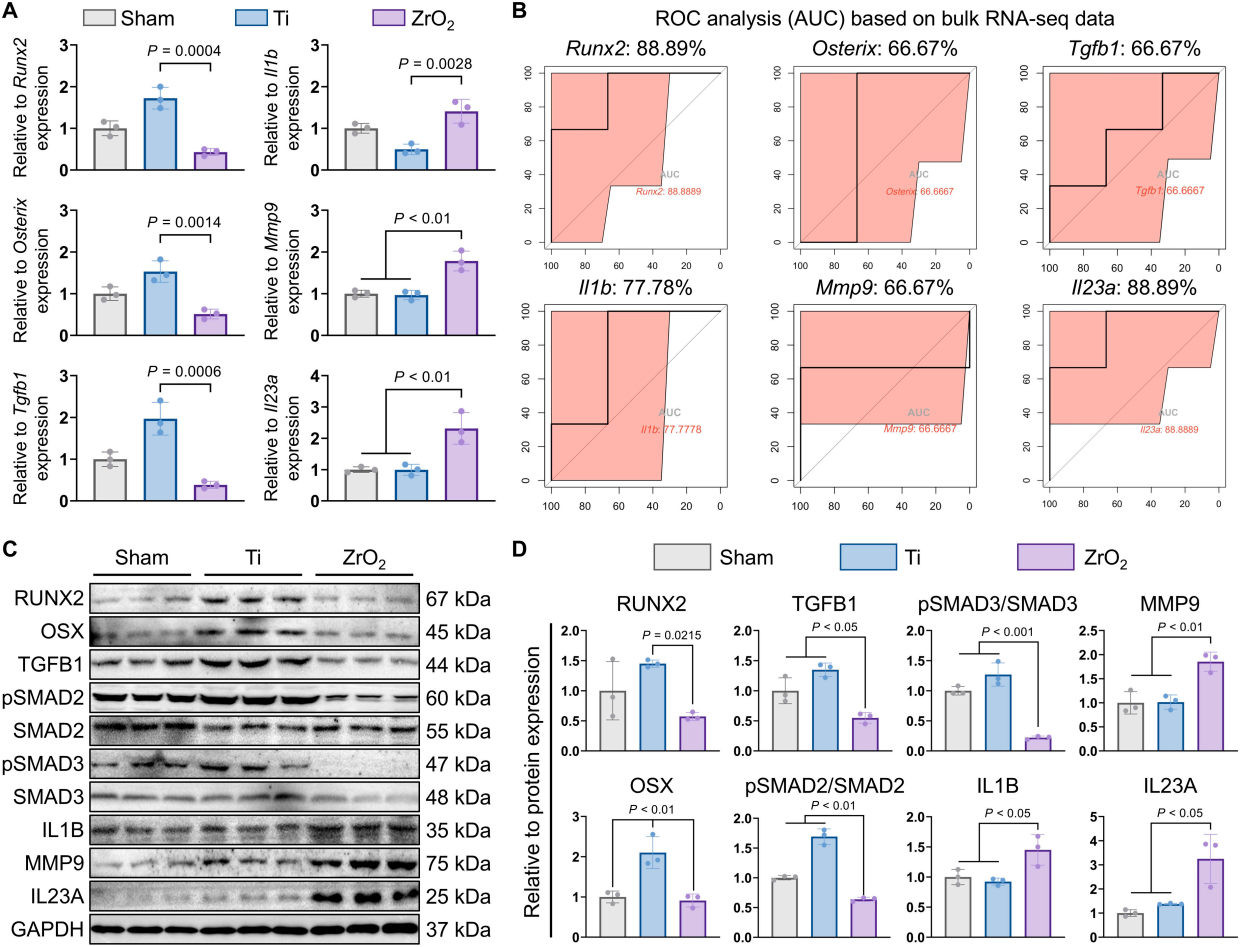
Ti and ZrO_2_ implants induce early osseointegration and immune inflammation. (A) Transcriptional levels of osteogenesis-related and immune-inflammatory genes. Osteogenic genes: *Runx2*, *Osterix*, and *Tgfb1*. Immune-inflammatory genes: *Il1b*, *Mmp9*, and *Il23a*. (B) Receiver operating characteristic (ROC) analysis based on bulk RNA-seq data. AUC, area under the curve. (C) Protein expression levels of osteogenesis-related and immune-inflammatory markers. Osteogenic proteins: RUNX2, OSX, TGFB1, pSMAD2/SMAD2, and pSMAD3/SMAD3. Immune-inflammatory proteins: IL1B, MMP9, and IL23A. (D) Statistical analysis of protein expression levels. Statistical significance was determined using one-way analysis of variance. RUNX2, RUNX family transcription factor 2; OSX, osterix; TGFB1, transforming growth factor beta 1; SMAD2, SMAD family member 2; SMAD3, SMAD family member 3; IL1B, interleukin 1 beta; MMP9, matrix metallopeptidase 9; IL23A, interleukin 23 subunit alpha.

To further validate the aforementioned analytical results, we examined the transcriptional levels of osteogenic genes (*Runx2*, *Osterix*, and *Tgfb1*) and immune-inflammatory-related genes (*Il1b*, *Mmp9*, and *Il23a*) in the sham, Ti, and ZrO_2_ groups. The results showed that the transcriptional levels of osteogenic genes were significantly higher in the Ti group compared to those in the ZrO_2_ group, while the opposite trend was observed for immune-inflammatory genes, which exhibited significant up-regulation in the ZrO_2_ group (Fig. 7A). Furthermore, receiver operating characteristic analysis based on bulk RNA-seq data was performed on the aforementioned genes, and their predictive efficacy was evaluated using area under the curve (AUC). The results indicated that the AUC values for these genes were all greater than 60%, suggesting good predictive performance (Fig. 7B).

Subsequently, proteins were extracted from the peri-implant bone-marrow tissue, and the expression levels of osteogenesis-related proteins (RUNX2 [RUNX family transcription factor 2], OSX [osterix], TGFB1 [transforming growth factor beta 1], pSMAD2/SMAD2 [phosphorylated SMAD family member 2/SMAD family member 2], and pSMAD3/SMAD3 [phosphorylated SMAD family member 3/SMAD family member 3]) were detected by Western blotting. Compared to that in the ZrO_2_ group, the expression of osteogenesis-related proteins was significantly up-regulated in the Ti group. Concurrently, the expression of immune-inflammatory-related proteins (IL1B [interleukin 1 beta], MMP9 [matrix metallopeptidase 9], and IL23A [interleukin 23 subunit alpha]) was also examined. The results showed significant up-regulation in the ZrO_2_ group, while no significant change was observed in the Ti group (Fig. 7C and D).

In addition, a meta-analysis of recent studies on bone–implant contact for Ti and ZrO_2_ bone implants revealed that in the implantation phase, Ti exhibits better bone–implant contact efficacy compared to ZrO_2_ (*P* = 0.008) (Fig. S6A and B). To corroborate the biological functions mediated by the COL1A1/SDC1 axis in Ti and the COL6A2/CD44 axis in ZrO_2_, we conducted cell-type-specific in silico knockout of the key cell communication genes *Col1a1* and *Col6a2* based on scRNA-seq data, which was subsequently complemented with artificial-intelligence-driven evolutionary analysis for functional inference (Fig. S7A). Notably, *Col1a1* knockout in Ti group cells elicited inflammatory activation, cell apoptosis, and growth inhibition (Fig. S7B). Conversely, *Col6a2* knockout in ZrO_2_ group cells promoted enhanced energy supply, ECM synthesis, and the establishment of repair homeostasis (Fig. S7C). These results collectively support our conclusions that Ti promotes ECM remodeling and activates a subsequent osteogenic microenvironment via TGF-β signaling, whereas ZrO_2_, despite its favorable aesthetics and biocompatibility, tends to activate inflammatory macrophages and promote the activation of immune-inflammatory pathways, thereby forming a peri-implant inflammatory microenvironment that impairs osseointegration.

## Discussion

The osteoimmune microenvironment forms a dynamic interface. Here, biomaterials, immune cells, and stromal components participate in complex cross talk, which substantially impacts the outcome of osseointegration [18,19]. Given the decisive early phase of bone regeneration, the present study investigated the early cellular dynamics and molecular mechanisms underlying the distinct osteoimmunological responses induced by Ti and ZrO_2_ implants via single-cell transcriptomics, computational analysis, and experimental validation [20]. Our findings revealed a fibroblast-centered regulatory axis (COL1A1/SDC1) that facilitates titanium-induced optimization of the bone-marrow microenvironment during the initial implantation phase, whereas ZrO_2_ implants elicit lymphoid-driven inflammatory activation.

### Ti and ZrO_2_ implants induce distinct bone-marrow microenvironments

This study identified distinct cellular responses to intramedullary femoral Ti and ZrO_2_ implants, enhancing the conventional understanding of material biocompatibility. Ti has long been considered the gold standard for orthopedic implants, a status underpinned by its exceptional corrosion resistance and biocompatibility [21–23]. However, our scRNA-seq data revealed that its superiority may instead stem from the active modulation of stromal–immune cross talk. Notably, the Ti-implanted microenvironment demonstrated remarkable preservation of physiological homeostasis, as evidenced by minimal deviation from sham-operated controls. This integration strategy is characterized by a concurrent increase in the number of progenitor/stem cells and a significant reduction in the number of lymphoid infiltrates compared with those in the ZrO_2_-implanted group. These findings indicate that Ti-based materials suppress early inflammatory cascades and promote progenitor/stem-cell retention—a dual mechanism critical for successful osseointegration.

The reduced peri-implant fibrosis observed with Ti implants in this study aligns with clinical reports comparing Ti with alternative materials and is mechanistically rooted in the distinct immunomodulatory properties of these implants [24,25]. Although previous studies have attributed this advantage to passive biocompatibility, our data demonstrate that Ti actively reprograms stromal cells to suppress fibrosis—a phenomenon that is causative rather than merely correlative. Specifically, our scRNA-seq trajectories showed that Ti-associated fibroblasts down-regulate fibrosis drivers such as α-SMA and up-regulate TGF-β, a known cytokine that promotes osteogenesis via the SMAD signaling pathway [26].

### Ti-based implants optimize bone-marrow microenvironment via fibroblastic COL1A1/SDC1 signaling

Our findings indicate that Ti-based implants optimize the bone-marrow microenvironment through fibroblast-mediated activation of the COL1A1/SDC1 signaling axis, which promotes ECM remodeling, stem-cell retention, and early osteogenic microenvironment. scRNA-seq analysis revealed that Ti implantation significantly reduced lymphoid populations and enriched progenitor and stem cells, indicating enhanced proliferative capacity and immune homeostasis. Fuzzy *C*-means clustering identified Ti-specific gene modules enriched in cell cycle regulation, energy metabolism (ATP-binding cassette transporters), and hyaluronan-driven ECM dynamics. Cell–cell interaction analysis identified fibroblasts as the primary orchestrators, with the COL1A1/SDC1 ligand–receptor pair exhibiting the strongest autocrine signaling. Subsequent experiments confirmed that Ti-induced fibroblasts coexpress and colocalize COL1A1 (a fibrillar collagen) and SDC1 (a heparan sulfate proteoglycan) at binding interfaces with high affinity. This interaction triggers the activation of the TGF-β pathway, leading to the activation of osteogenesis-related SMAD signaling, as well as the up-regulation of the osteogenic proteins RUNX2 and OSX, thereby facilitating the formation of an early osteogenic microenvironment [27,28].

Fibroblasts have emerged as pivotal regulators of early tissue regeneration, orchestrating ECM remodeling and immune–stromal cross talk during the critical inflammatory-to-reparative phase transition [29,30]. As a readily accessible cell type, fibroblasts have also gained attention as a promising alternative to mesenchymal stem cells in regenerative medicine, particularly due to their immunomodulatory properties [31,32]. For instance, Layton et al. [33] have employed single-cell analysis to investigate cellular heterogeneity in human fibrotic tissues, revealing that the identification of immune-regulatory ICAM1^+^ fibroblasts highlights their role in immune-cell recruitment and tissue repair. These fibroblasts can suppress lymphocyte proliferation and modulate immune-cell phenotypes, thereby acting as key regulatory nodes in the immune network [32,34]. Our findings are consistent with this paradigm shift, demonstrating that Ti implants actively exploit fibroblast plasticity to establish a pro-regenerative niche. More importantly, the activation of the COL1A1/SDC1 signaling axis by Ti implants reveals a novel mechanism of ECM remodeling. COL1A1, a key component of type I collagen, is crucial for ECM structure and function [35], whereas SDC1, a heparan sulfate proteoglycan, serves as a coreceptor for various growth factors and cytokines [36]. Their interaction, as demonstrated in this study, activates downstream signaling pathways, such as TGF-β signaling, which plays a pivotal role in ECM regulation and immune homeostasis [37,38]. This finding aligns with recent research emphasizing the dynamic role of the ECM in tissue repair and regeneration, wherein ECM-derived signals can direct stem-cell behavior and modulate immune responses [39,40].

### ZrO_2_ implants induce an inflammatory bone-marrow microenvironment via fibroblastic COL6A2/CD44

This study demonstrated that ZrO_2_ implants induce fibrosis-associated inflammation bone-marrow niches via COL6A2/CD44-mediated fibroblast–macrophage cross talk. Bioinformatic analyses revealed ZrO_2_-specific activation of innate immunity, ECM–receptor interactions, and collagen organization pathways, accompanied by stromal–immune dysregulation and elevated myeloid populations. Cell–cell interaction mapping identified fibroblasts as primary signal senders and macrophages as dominant receivers, with collagen-related signaling as the core pathway. Ligand–receptor profiling pinpointed fibroblast-derived COL6A2 and macrophage CD44 as the strongest interacting pair. Structural and spatial analyses confirmed high-affinity binding between the domain of COL6A2 and the hyaluronan-binding region of CD44, with COL6A2^+^ fibroblasts (S100A4^+^) juxtaposed to CD44^+^ macrophages (CD68^+^) exclusively in ZrO_2_ tissues. These findings establish the COL6A2/CD44 axis as a ZrO_2_-specific driver of macrophage activation and ECM remodeling, in contrast to the regenerative COL1A1/SDC1 axis of Ti.

The identification of COL6A2/CD44 as a ZrO_2_-specific fibro-inflammatory axis redefines our understanding of implant biocompatibility, highlighting how material properties govern stromal–immune metabolic symbiosis. COL6A2, a basement-membrane collagen protein, is increasingly recognized as a critical factor in fibrosis [41,42], where its extracellular deposition alters tissue stiffness to activate CD44-mediated pro-inflammatory signaling in macrophages [43,44]. This mechanism is particularly pronounced in ZrO_2_ implants, where material-specific surface properties synergize with biomechanical cues to amplify COL6A2/CD44-mediated inflammation [45]. In fact, ZrO_2_ surfaces exhibit enhanced hydrophobicity compared with titanium, promoting the adsorption of IgG and complement C1q proteins [46]. This immunoglobulin–complement complex triggers activation of the classical complement pathway, generating anaphylatoxins (C3a/C5a) that sensitize macrophages to COL6A2/CD44 signaling [46]. Concurrently, ZrO_2_-derived micro/nanoparticles—although less cytotoxic than titanium particles—induce chronic mechanical stress in cells, up-regulating cytokine expression [47]. The resulting increase in collagen VI deposition creates a fibrotic niche with elevated tissue stiffness, further enhancing pro-inflammatory activity in macrophages [48,49]. Notably, the surface physicochemical properties of ZrO_2_ modulate these interactions. Rough surfaces exacerbate protein adsorption and complement activation, whereas hydrophilic modifications may attenuate these effects by reducing COL6A2 secretion and CD44 clustering [48,50].

When interpreting these material-specific biological outcomes, we considered the impact of surface topography and emphasized the core role of intrinsic physicochemical properties: standardized polishing controlled the key roughness parameters (e.g., Sa and Sq) of Ti and ZrO_2_ implants to comparable levels, minimizing topographic confounding effects. Thus, observed differences in immune–stromal interactions and signaling pathways are likely driven by intrinsic chemical differences—especially ZrO_2_’s unique interfacial traits as a ceramic. Notably, its high hydrophobicity induces conformational changes in proteins (e.g., fibrinogen) to expose integrin-binding sites, prompting fibroblasts to up-regulate COL6A2 via integrin signaling; its enhanced protein adsorption also enriches specific cytokines, creating a microenvironment that drives macrophage polarization toward a pro-inflammatory phenotype via CD44. This mechanistic link confirms that intrinsic material chemistry plays a decisive role in early osteoimmunological programming. Building on this, WGCNA identified a Ti-associated green module (correlation coefficient = 0.64) and a ZrO_2_-associated turquoise module (correlation coefficient = 0.93), while LASSO regression further confirmed *Col1a1* as the key feature gene in the Ti group and *Cd44* as the most significant one in the ZrO_2_ group—providing transcriptional evidence for the functional importance of the COL1A1/SDC1 and COL6A2/CD44 axes we previously characterized.

This study demonstrated that Ti- and ZrO_2_ implants elicit distinct osteoimmune responses, primarily through stromal–immune interactions. An integrated multi-omics methodology combining whole-exome sequencing and deep proteomic profiling enables the construction of proteogenomic maps [51]. By synergizing with nanoprobe-based technologies for macrophage phenotyping, this approach will facilitate data-driven optimization of biomaterial architectures, thereby advancing precision-matched clinical applications through materials science innovations [52]. Furthermore, exploring the impact of implant surface topography, including nanoscale features, on modulating stromal–immune cross talk may lead to innovative biomaterial design strategies.

By focusing on the 3-d postimplantation period—a critical early phase of inflammation and tissue initiation—the initial immune microenvironment events that drive subsequent differences in osseointegration can be revealed. Although long-term in vivo evaluations were not performed, the signaling functions we identified are highly consistent with known pathophysiological processes that determine the long-term success or failure of implants. Studies have shown that early fibrous encapsulation and chronic inflammation are important factors leading to poor osseointegration and late-stage implant loosening [53]. Conversely, stem-cell enrichment and an osteogenic microenvironment state are considered ideal prerequisites for achieving rapid and stable osseointegration [54]. Therefore, targeted intervention of key signaling axes, such as COL1A1/SDC1 and COL6A2/CD44, during the early stage may regulate subsequent tissue healing trajectories, thereby improving long-term osseointegration outcomes of the implant. Our experimental design incorporated considerations for specific technical limitations, including constrained cellular yield from fluorescence-activated cell sorting, the inherent reductionism of in vitro co-culture models, and the methodological complexities associated with hard-tissue histomorphometric analysis in long-term osseointegration studies. To address these, we employed a complementary multi-level validation strategy integrating spatial, transcriptional, and phenotypic analyses. Furthermore, we constructed a virtual cellular model to computationally simulate *Col6a2* knockdown within the ZrO_2_-implanted niche. This in silico perturbation effectively attenuated the local pro-inflammatory response, corroborating our proposed mechanism. While providing supportive evidence, such computational simulations cannot fully replace direct in vivo validation. Consequently, future studies employing genetic models—such as fibroblast-specific *Col6a2* knockout—will be essential to establish definitive causal evidence for this identified signaling axis.

## Conclusion

This study demonstrates that Ti-based implants induce a favorable bone-marrow microenvironment by activating the COL1A1/SDC1 signaling pathway in fibroblasts, promoting cell proliferation, energy metabolism, ECM remodeling, and early osteogenesis. In contrast, ZrO_2_ implants induce a fibro-inflammatory niche through the COL6A2/CD44 axis, stimulating inflammatory macrophage activation. These material-specific immunomodulatory effects highlight the critical role of biomaterial properties in shaping host responses. Importantly, the mechanistic insights gained here provide a framework for guiding the osteogenic functionalization of ZrO_2_ implants and inform the rational design of next-generation immunomodulatory biointerfaces.

## Materials and Methods

### Preparation of materials

Commercial 3 mol% yttria-stabilized tetragonal zirconia polycrystal (Cercon, Dentsply Sirona) was used in the form of presintered blocks. For in vitro studies, discs (10 mm × 10 mm × 1 mm) were machined; for in vivo studies, cylindrical rods (1 mm × 1 mm × 2.5 cm) were prepared. All ZrO_2_ substrates were sequentially polished using silicon carbide (SiC) sandpaper with grit sizes ranging from 400 to 2,000 to achieve a mirrorlike surface. Subsequently, they were ultrasonically cleaned in acetone, 75% ethanol, and deionized water (20 min each) to remove organic residues and particulate contaminants. Prior to biological experiments, the substrates were sterilized by autoclaving, dried, and exposed to ultraviolet irradiation for 3 h. For comparison, Ti substrates were similarly polished using the same SiC grit sequence, followed by identical ultrasonic cleaning and sterilization procedures.

### Surface characterization

The surface morphologies of Ti and ZrO_2_ samples were examined using a field emission SEM (JSM-7001F, JEOL, Japan) operated at an accelerating voltage of 10 kV. Elemental distribution on the sample surfaces was analyzed using energy-dispersive x-ray spectroscopy (QX200, Bruker Optics, Germany). The surface roughness of each specimen was measured using a noncontact confocal laser scanning microscope (VK-X3000 series, KEYENCE, Germany) at 20× magnification. Surface wettability was assessed using a contact-angle goniometer (JC2000DF, Powereach, China) via the pendant-drop method. For each sample, 3 fields of view were captured, and 5 parallel samples were analyzed.

### Cell culture

The L929 fibroblast cells were cultured at 37 °C with 5% CO_2_. For experimental purposes, cells from passages 4 to 7 were seeded at a density of 5 × 10^5^ cells per well into 6-well plates containing either material discs (Ti and ZrO_2_) or extraction media, followed by 72-h incubation. Subsequent analyses included evaluation of cellular viability/mortality status and assessment of cell-adhesion characteristics. All in vitro experiments were performed in triplicate across 3 independent experiments to ensure statistical reliability.

### Protein adsorption

Material discs (Ti and ZrO_2_, *n* = 3) were incubated in a 5 mg/ml bovine serum albumin solution in phosphate-buffered saline (PBS) at 37 °C for 4 h. After incubation, the samples were gently rinsed with PBS to remove any residual unbound proteins. The concentration of adsorbed proteins was quantified using a microplate reader (A51119500C, Thermo Fisher Scientific, USA) at 525 nm. A standard curve of bovine serum albumin was established, and the amount of protein adsorbed onto the surfaces of Ti and ZrO_2_ was calculated accordingly.

### Calcein-acetoxymethyl ester/propidium iodide staining

Ti and ZrO_2_ material discs were immersed in Dulbecco’s modified Eagle medium (DMEM) supplemented with 10% fetal bovine serum within 6-well culture plates. Following a 72-h static incubation period under standard cell culture conditions (37 °C, 5% CO_2_, 95% humidity), the resultant extracts were collected under laminar flow biosafety cabinet conditions. Fluorescence-based viability analysis of L929 cells (cells were cultured with Ti and ZrO_2_ extracts for 72 h) was performed using a calcein-acetoxymethyl ester (calcein-AM)/propidium iodide (PI) double-staining kit (CA1630, Solarbio, UK), according to the manufacturer’s instructions and previously established protocols [55]. In this assay, live cells, stained with calcein-AM, and dead cells, stained with PI, emit green and red fluorescence, respectively.

### Cell-adhesion assay

L929 cells were seeded at a density of 5 × 10^5^ cells per well into 6-well plates containing material discs that had been pre-immersed in DMEM supplemented with 10% fetal bovine serum. The cells were cultured under standard conditions (37 °C, 5% CO_2_, 95% humidity) for 72 h. After incubation, the cell morphology and distribution around the materials were observed using an optical microscope.

Subsequently, the material discs were carefully removed and fixed with glutaraldehyde fixative for electron microscopy for 20 min. The attached cells were then dehydrated through a graded ethanol series and allowed to air-dry at room temperature for 24 h. Finally, the cell adhesion on the material surfaces was examined and imaged using SEM.

### Animal model of intramedullary femoral implantation

All animal procedures were approved by the Institutional Animal Care and Use Committee and the Ethics Committee of Guangzhou Medical University (Approval No. GY2023-721) and conducted in accordance with institutional guidelines.

Based on previous studies, 18 male Sprague–Dawley rats (300 g, 8 to 12 weeks old) were anesthetized via intraperitoneal injection of Zoletil 5 (30 mg/kg) and xylazine hydrochloride (5 mg/kg) [56,57]. Cylindrical material rods (Ti and ZrO_2_, dimensions described above) were implanted into the femoral medullary cavity via a drilled channel through the distal femoral condyle, ensuring contact with both cancellous/cortical bone and marrow tissue. For the sham group, cylindrical material rods were briefly inserted and then immediately withdrawn without retention in the implantation site. The surgical site was sutured and disinfected under sterile conditions. On postoperative day 3, the rats were euthanized (rats were euthanized via the CO_2_ gradual displacement method, adhering to ARRIVE guidelines to ensure rapid unconsciousness and minimize distress), and the femurs and bone marrow surrounding the implants were harvested for analysis.

### Bone-marrow sample collection

On postoperative day 3, following euthanasia, bone marrow adjacent to the implant was harvested for scRNA-seq. To ensure precise sampling of the tissue microenvironment directly influenced by the implant, a standardized protocol was employed. The femur was exposed and stabilized. Using a fine-gauge (e.g., 22G to 25G) bone-marrow aspiration needle under direct visualization with a surgical microscope (Leica M80, 8× to 10× magnification, Germany), the needle tip was positioned at the implant–marrow interface. The marrow tissue residing within a defined distance of approximately 1 mm from the implant surface was gently flushed with sterile PBS using a syringe. The resulting marrow suspension was immediately collected into pre-chilled RNase-free tubes and placed on ice to preserve cell viability and RNA integrity until further processing. This procedure was performed consistently for all animals in the study.

### Single-cell RNA sequencing

For scRNA-seq of the harvested bone marrow (each group comprised 3 independent biological specimens), the Gel Bead-in-Emulsion (GEM) Single Cell 3′ Reagent Kit was used according to the manufacturer’s instructions. Specifically, single-cell GEMs were generated using the kit, incorporating cell barcodes into individual droplets. This step was followed by post-GEM reverse transcription cleanup and complementary DNA amplification. A 3′ gene expression library was then constructed before sequencing. The MobiVision software pipeline (version 1.1, MobiDrop) was employed to demultiplex cell barcodes. Sequence reads were aligned to the reference genome and transcriptome using the STAR aligner. When necessary, read downsampling was performed to normalize data across samples. This process yielded a gene-by-cell count matrix, enabling a comprehensive analysis of the transcriptional landscape within the reparative tissue.

### scRNA-seq data processing and analysis

The R package Seurat was used for quality control, dimensionality reduction, and clustering of the scRNA-seq data [58]. Cells with a gene count between 200 and 6,000 and a mitochondrial gene content below 25% were considered high quality and included in subsequent analyses. Following normalization, highly variable genes were identified using the FindVariableFeatures() function. Principal component analysis and Uniform Manifold Approximation and Projection were then conducted for dimensionality reduction and visualization. Cell clustering and annotation were based on canonical marker genes. Differential gene expression analysis was conducted using the FindAllMarkers() function in Seurat. Intercellular communication between distinct cell subpopulations was inferred using the CellChat package. KEGG and GO enrichment analyses of DEGs were visualized using the SangerBox online platform.

For the bulk RNA-seq data, the limma package was employed to identify DEGs with a cutoff of |logFC| > 1.5 and *P* < 0.05 [59,60]. The WGCNA package was used to perform WGCNA [61]. LASSO regression analysis was performed using the lars package to further filter DEGs [62]. Gene pseudotime trajectory analysis was performed using the Slingshot package to explore temporal gene expression patterns [63]. The additional analytical procedures are incorporated into the Supplementary Materials section.

### Histological analyses

The decalcified bone tissues were cut into 5-μm-thick sections. The sections were then deparaffinized in xylene and rehydrated through an ethanol gradient (100%, 95%, 80%, and 70%) to water. After thorough rinsing, nuclei were stained with hematoxylin, differentiated in acid alcohol, and blued in alkaline water. Following counterstaining with eosin, the sections were dehydrated in a graded ethanol series, cleared in xylene, and sealed with neutral resin. Finally, observation and imaging were performed under a light microscope.

For immunofluorescence costaining, a multiplex fluorescence immunohistochemistry mouse/rabbit kit (pH 9.0) (RS0037, ImmunoWay, USA) was used according to the manufacturer’s instructions. The following antibodies were applied sequentially: CD44 (Bioss, bsm-54767R, 1:300, China), CD68 (Proteintech, 66231-2-Ig, 1:500, China), CD206 (Bioss, bsm-55604R, 1:300, China), NOS2 (Bioss, bs-0162R, 1:300, China), COL6A2 (Bioss, bs-13963R, 1:300, China), S100A4 (Bioss, bs-3759R, 1:300, China), COL1A1 (Bioss, bs-10423R, 1:300, China), and SDC1 (Bioss, bs-1309R, 1:300, China), followed by nuclear counterstaining with 4′,6-diamidino-2-phenylindole. ImageJ was used to analyze the colocalization of staining markers.

### Western blotting

Total protein was extracted from the peri-implant bone-marrow tissue using radioimmunoprecipitation assay lysis buffer (P0013B, Beyotime, China). The lysates were sonicated, collected, and centrifuged at 4 °C (13,400 rpm, 15 min). The proteins were separated and transferred onto polyvinylidene fluoride membranes (P0690, Beyotime, China) using 10% sodium dodecyl sulfate–polyacrylamide gel electrophoresis gels. The membranes were blocked with 5% nonfat milk for 1 h, followed by overnight incubation at 4 °C with the following primary antibodies: RUNX2 (Bioss, bs-1134R, 1:1,000, China), OSX (Bioss, bs-25532R, 1:1,000, China), pSMAD2 (Bioss, bs-3419R, 1:1,000, China), pSMAD3 (Bioss, bs-3425R, 1:1,000, China), IL1B (Bioss, bs-0812R, 1:1,000, China), MMP9 (Bioss, bsm-54040R, 1:3,000, China), IL23A (Bioss, bs-1193R, 1:1,000, China), NOS2 (Bioss, bs-0162R, 1:2,000, China), CD206 (Bioss, bsm-55604R, 1:1,000, China), TGFB1 (Proteintech, 81746-2-RR, 1:1,000, China), SMAD2 (Proteintech, 12570-1-AP, 1:3,000, China), and SMAD3 (Proteintech, 30130-1-AP, 1:2,000, China). After 3 washes with TBST buffer, the membranes were incubated with horseradish peroxidase-conjugated secondary antibodies at room temperature for 1 h. The bound antibodies were then detected using an Odyssey infrared imaging system (Odyssey DLx, LI-COR). Band intensities were analyzed with the ImageJ software (version 1.8.0).

### Statistical analysis

All values are presented as mean ± standard deviation. The Student *t* test was employed to assess the statistical significance between 2 groups. Statistical analysis was performed using GraphPad Prism 9.5 (GraphPad Software, CA, USA). Differences were considered statistically significant at *P* < 0.05.

## Data Availability

Data will be made available on request.
