# Supplementary material for: Mapping Immune-Inflammatory Niches on Zirconia Bone Implants: Single-Cell Transcriptomic Profiling
Source: Research (Wash D C). 2026 Mar 10;9:1162. doi: 10.34133/research.1162 (PMC12972508; doi:10.34133/research.1162)
Supplement: Supplementary 1 — Supplementary Materials and Methods Table S1 Figs. S1 to S7 [file research.1162.f1.docx]

**Mapping Immune-Inflammatory Niches on Zirconia Bone Implants: Single-Cell Transcriptomic Profiling**

Jiannan Zhou ^1^, An Li ^2^, Jiahao Chen ^3^, Jingtao Dai ^4^, Wentai Zhang ^5,^ *, Zhilu Yang ^5,^ *, Ping Li ^1,^ *

1. School and Hospital of Stomatology, Guangdong Engineering Research Center of Oral Restoration and Reconstruction & Guangzhou Key Laboratory of Basic and Applied Research of Oral Regenerative Medicine, Guangzhou Medical University, Guangzhou, China.
2. Department of Periodontology, Stomatological Hospital, School of Stomatology, Southern Medical University, Guangzhou, China.
3. Department of Prosthodontics, Geriatric Dentistry and Craniomandibular Disorders, Charité-Universitätsmedizin Berlin, corporate member of Freie Universität Berlin and Humboldt-Universität zu Berlin, Berlin, Germany.
4. Department of Orthodontics, Stomatological Hospital, School of Stomatology, Southern Medical University, Guangzhou, China.
5. The Tenth Affiliated Hospital, Southern Medical University (Dongguan People's Hospital), Dongguan, Guangdong, 523059, China

These authors contributed equally: Wentai Zhang, Zhilu Yang, Ping Li

* Address correspondence to: School and Hospital of Stomatology, Guangdong Engineering Research Center of Oral Restoration and Reconstruction & Guangzhou Key Laboratory of Basic and Applied Research of Oral Regenerative Medicine, Guangzhou Medical University, Guangzhou, China.

Email: [zhangwentai@smu.edu.cn](mailto:zhangwentai@smu.edu.cn), [zhiluyang1029@smu.edu.cn](mailto:zhiluyang1029@smu.edu.cn), [pingli@gzhmu.edu.cn](mailto:pingli@gzhmu.edu.cn)

**1. Supplementary Materials and Methods**

**1.1 Single-cell communication analysis**

The analysis of intercellular communication networks in single cells was conducted using the CellChat pipeline. Initially, raw single-cell transcriptomic data was standardized (via SCTransform method) and batch-corrected (using Harmony algorithm) based on "Seurat". After obtaining cell subsets through hierarchical clustering, cell types were annotated by referencing a rat bone marrow atlas dataset. For cell communication analysis, the CellChat platform was employed, which utilized its built-in CellChatDB database to quantify interaction strengths between cell populations through a communication probability model. The model was optimized by maximum likelihood estimation, with interaction scores calculated from the geometric mean of ligand and receptor expression levels. To control false positives, significant pathways were screened using 1,000 permutation tests (FDR-corrected threshold *p* < 0.05), and hub cell populations were identified based on network topology analysis (betweenness centrality, closeness centrality).

**1.2 Time series gene expression trend analysis**

The elucidation of time-dependent gene expression patterns was achieved through fuzzy c-means clustering implemented in the "Mfuzz" package. After log2 transformation of raw expression data (TPM values), low-variance genes (standard deviation < 0.5 across time points) were filtered out. The retained genes were used for downstream Gene Ontology (GO) and Kyoto Encyclopedia of Genes and Genomes (KEGG) analyses. Data standardization was performed using the standardise function in Mfuzz, which applied z-score normalization to each gene to eliminate scale differences. A gene-cluster membership threshold of > 0.7 was set to select high-confidence member genes. Cluster stability was assessed via 100 Bootstrap resamplings (Jaccard similarity > 0.85). Functional annotation of differential trend clusters employed a dual strategy: GO and KEGG enrichment analyses (FDR-corrected threshold *p*_adj_ < 0.05) were conducted based on cluster core genes (top 20% membership genes). Pathway-time association network plots were generated using enrichplot to quantify the intensity of pathway activity changes over time. Kinetic curves of key trend modules were plotted using ggplot2 with smoothed spline fitting, and cluster heatmaps were visualized using ComplexHeatmap sorted by time gradients.

**1.3 Bulk RNA-seq and data analysis**

Total RNA was extracted using TRIzol reagent according to the manufacturer's instructions. RNA purity and quantity were evaluated using a spectrophotometer (NanoDrop 2000, Thermo Scientific), while RNA integrity was assessed using a bioanalyzer (Agilent 2100, Agilent Technologies, Santa Clara). Transcriptomic libraries were constructed using the VAHTS Universal V6 RNA-seq Library Prep Kit according to the provided protocol, followed by sequencing on the Illumina Novaseq 6000 platform to generate 150 bp paired-end reads. Raw reads in fastq format were processed using fastp software to eliminate low-quality reads and provide high-quality reads for downstream data analysis. HISAT2 software was used for reference genome alignment, and gene expression levels (FPKM) were quantified. Read counts for each gene were generated using HTSeq count.

**1.4 Differential expression genes analysis**

Differential analysis of bulk RNA-seq datasets was performed using the R package “limma” to identify differentially expressed genes (DEGs) among Sham, Ti, and ZrO_2_ groups. The expression profile dataset was first log_2_-transformed, followed by multivariate linear regression analysis using the lmFit function. Then, the eBayes function was used to calculate moderated t-statistics, moderated F-statistics, and log-odds of differential expression, with standard errors adjusted using empirical Bayes to a common value. Heatmaps of DEGs were generated using the "pheatmap" package, and volcano plots were generated using the "ggplot2" package.

**1.5 Functional enrichment analysis**

The Database for Annotation, Visualization, and Integrated Discovery (DAVID, *http://david.abcc.ncifcrf.gov/*) is an online database that provides tools for classifying gene functions and assessing their biological roles. To investigate the functions of DEGs, GO and KEGG enrichment analyses were performed using the DAVID database. A statistically significant *p* < 0.05 cutoff was adopted to determine significant enrichments.

**1.6 Quantitative Real-Time PCR**

Total RNA was extracted from peri-implant tissues using a Trizol reagent kit. The RNA was reverse-transcribed into cDNA using the PrimeScript™ RT reagent kit (Takara Clontech, Kyoto, Japan). RT-qPCR was performed on a CFX Connect Real-Time System (BioRad, California, USA) using the SuperReal PreMix Plus (SYBR Green) kit (Takara Clontech, Kyoto, Japan). The thermal cycling protocol consisted of an initial step at 95°C for 5 min, followed by 40 cycles of denaturation at 95°C for 10 s, and annealing/extension at 60°C for 30 s. The mRNA expression levels were normalized to those of GAPDH. The primers used for RT-qPCR are listed in Supplementary Table 1.

**1.7 Receiver operating characteristic analysis**

Receiver Operating Characteristic (ROC) analysis was performed using the "pROC" package in R to evaluate the diagnostic performance of candidate genes. The area under the ROC curve (AUC) was used to determine the sensitivity of SS diagnostic genes in the dataset.

**1.8 Weighted gene co-expression network analysis**

The “WGCNA” package was used to construct co-expression networks and identify biologically significant gene modules associated with target traits. After preprocessing raw gene expression data, low-expression genes (average expression across samples < 0.1) were removed, and outlier samples were excluded via hierarchical clustering. A signed adjacency matrix was generated using the soft thresholding algorithm (soft threshold power *β* = 6, optimized by the pickSoftThreshold function to meet the scale-free topology criterion R^2^ > 0.80). Subsequently, the Topological Overlap Matrix (TOM) was calculated to reduce false correlation interference, and gene modules were identified by dynamic tree cutting. Module-trait associations were quantitatively evaluated by calculating the Pearson correlation between module eigengenes and phenotypic variables. Further analysis of the correlation between Gene Significance (GS) and Module Membership (MM) was performed to obtain Ti- and ZrO_2_-related gene modules.

**1.9 Lasso regression analysis**

Variable selection and regularization were performed using Lasso regression implemented in the “glmnet” package. Prior to modeling, continuous outcome variables were standardized (z-score normalization) to ensure coefficient comparability. Subsequently, regression analyses were conducted on the intersection genes (DEGs and WGCNA) of Ti and ZrO_2_ to determine the status of ligand-receptor genes.

**1.10 Virtual cell gene knockout**

We employed the “scTenifoldKnk” (*https://CRAN.R-project.org/package=scTenifoldKnk*) computational framework to perform in silico gene knockout analysis and predict the system-level transcriptional outcomes of target gene perturbation. First, a single-cell gene regulatory network was constructed using wild-type scRNA-seq data. Subsequently, the framework simulated the in silico knockout of a specific gene within this network and generated a ranked list of genes based on their predicted regulatory responsiveness to the knockout. Finally, the set of responsive genes identified from perturbing the target gene was subjected to functional enrichment analysis using the DAVID database to infer the potential biological functional alterations resulting from the specific gene knockout.

**2. Supplementary Table**

**Supplementary Table 1.** Primer sequences used for RT-qPCR.

| Gene Symbol | Species | Biotype | Primer | Sequence (5′ to 3′) |  |
| --- | --- | --- | --- | --- | --- |
| *Runx2* | Rat | mRNA | Forward | AGCAGTATTTACAACAGAGGGC |  |
|  |  |  | Reverse | CTGCACTGAAGAGGCTGTTTG |  |
| *Osterix* | Rat | mRNA | Forward | GGTCCTGGCAACACTCCTAC |  |
|  |  |  | Reverse | AAGAGGTGGGGTGCTGGATA |  |
| *Tgfb1* | Rat | mRNA | Forward | CTGCTGACCCCCACTGATAC |  |
|  |  |  | Reverse | AGCCCTGTATTCCGTCTCCT |  |
| *Il1b* | Rat | mRNA | Forward | CCTATGTCTTGCCCGTGGAG |  |
|  |  |  | Reverse | CACACACTAGCAGGTCGTCA |  |
| *Il23a* | | Rat | mRNA | Forward | CGGAAGCTCTGTAACTGCCT |
|  | |  |  | Reverse | CAGCTTTGTCACAGGTCGGT |
| *Mmp9* | | Rat | mRNA | Forward | AAACCTCCAACCTCACGGAC |
|  | |  |  | Reverse | TGGCCTTTAGTGTCTCGCTG |
| *Nos2* | | Rat | mRNA | Forward | TGCTTCTGTGCTAATGCGGA |
|  | |  |  | Reverse | CGCTTCCGACTTTCCTGTCT |
| *Cd206* | | Rat | mRNA | Forward | GGGTTCACCTGGAGTGATGG |
|  | |  |  | Reverse | ATTGTCTTGAGGAGCGGGTG |

**3. Supplementary Figure**


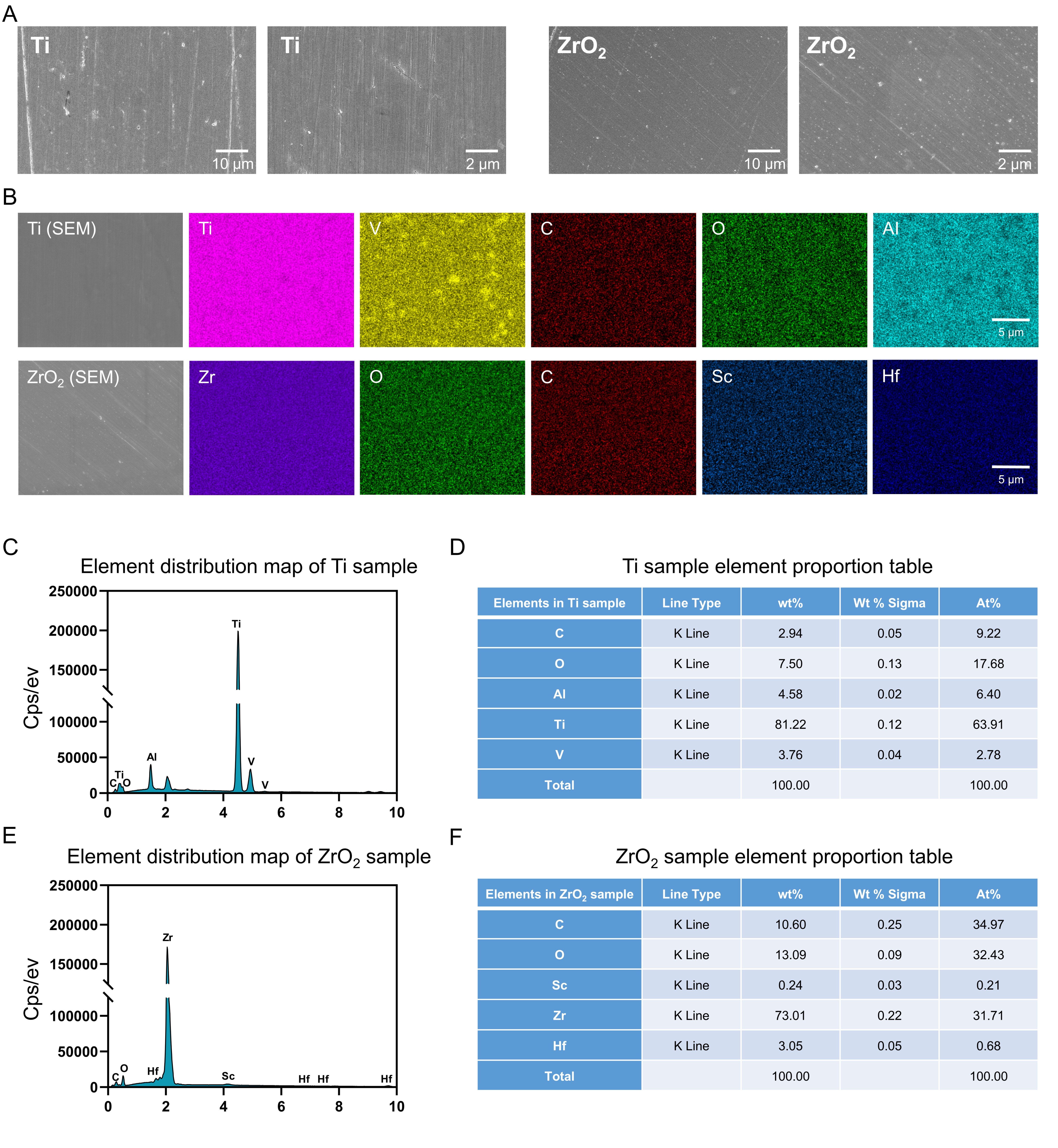


**Fig. S1** Characterization of Ti and ZrO_2_ materials. (A) SEM images of the material surfaces. Scale bars: 10 μm and 2 μm. (B) Energy-dispersive X-ray spectroscopy (EDS) element mapping of specific regions on material surfaces. Scale bars: 5 μm. (C, D) Elemental composition of specific regions on the Ti surface detected by EDS. (E, F) Elemental composition of specific regions on the ZrO_2_ surface detected by EDS.


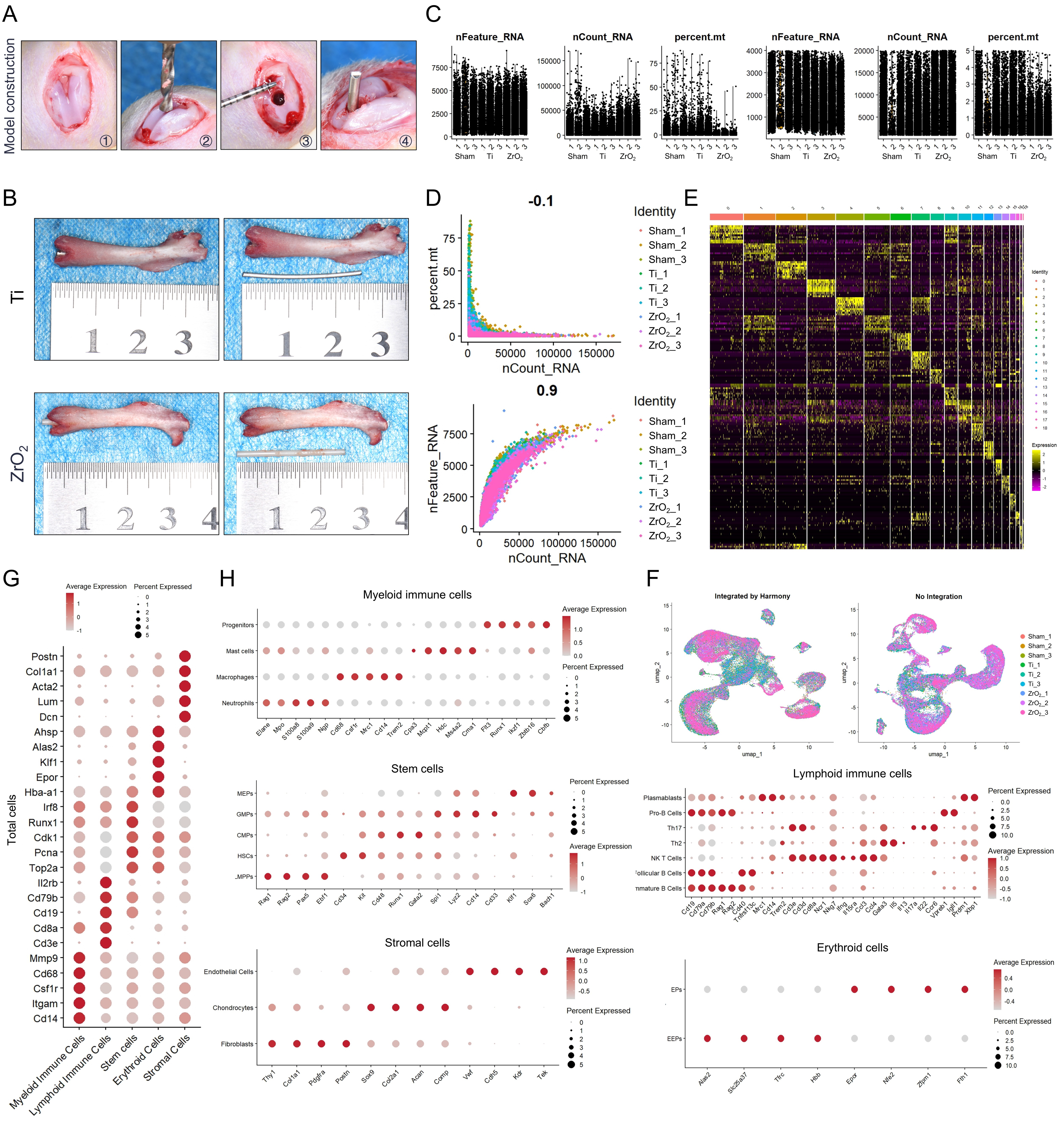


**Fig. S2** Establishment of the animal implantation model and integration analysis of scRNA-seq datasets. (A, B) Workflow of the animal implantation model and post-implantation material images. (C, D) Preprocessing of the scRNA-seq dataset and gene-sample association analysis. (E) Clustering analysis of highly variable genes within cell populations. (F, G) Identification of classic genes for cell type classification and their subpopulations. (H) Cell distribution before and after batch correction and dataset integration using Harmony in scRNA-seq analysis.

**
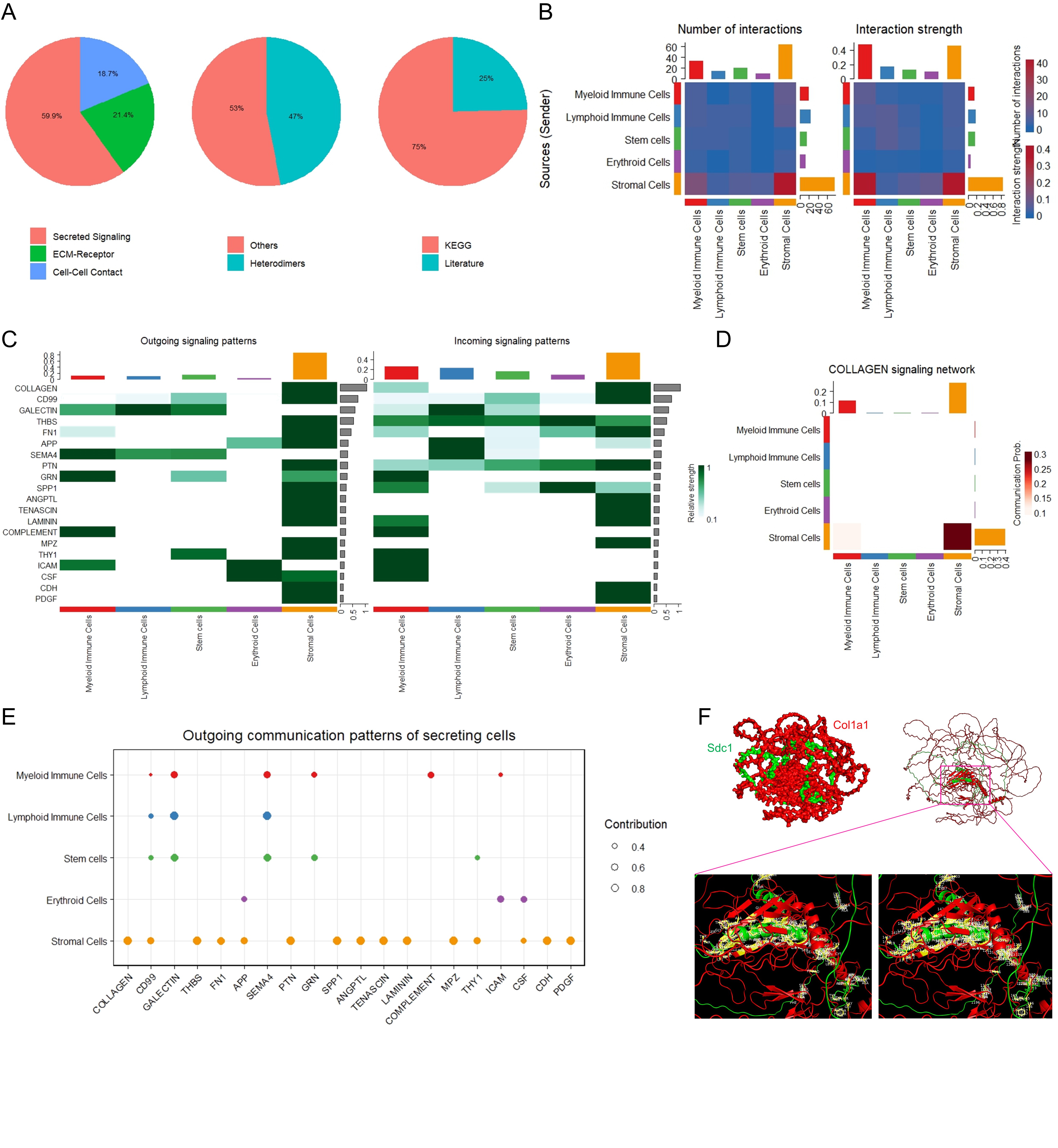
**

**Fig. S3** Intercellular crosstalk and ligand-receptor pair prediction in cells surrounding Ti implants. (A) Proportions of autocrine/paracrine signaling interactions, ECM-receptor interactions, and cell-cell contact interactions in the CellChat interaction database. (B) Heatmap visualization of intercellular interaction counts and strengths. (C) Visualization of Outgoing-Incoming signaling patterns and interaction strengths. (D) Analysis of intercellular interaction strengths in the COLLAGEN signaling network. (E) Outgoing communication patterns of secreting cells. (F) Molecular dynamics simulation and binding site prediction of COL1A1 and SDC1.


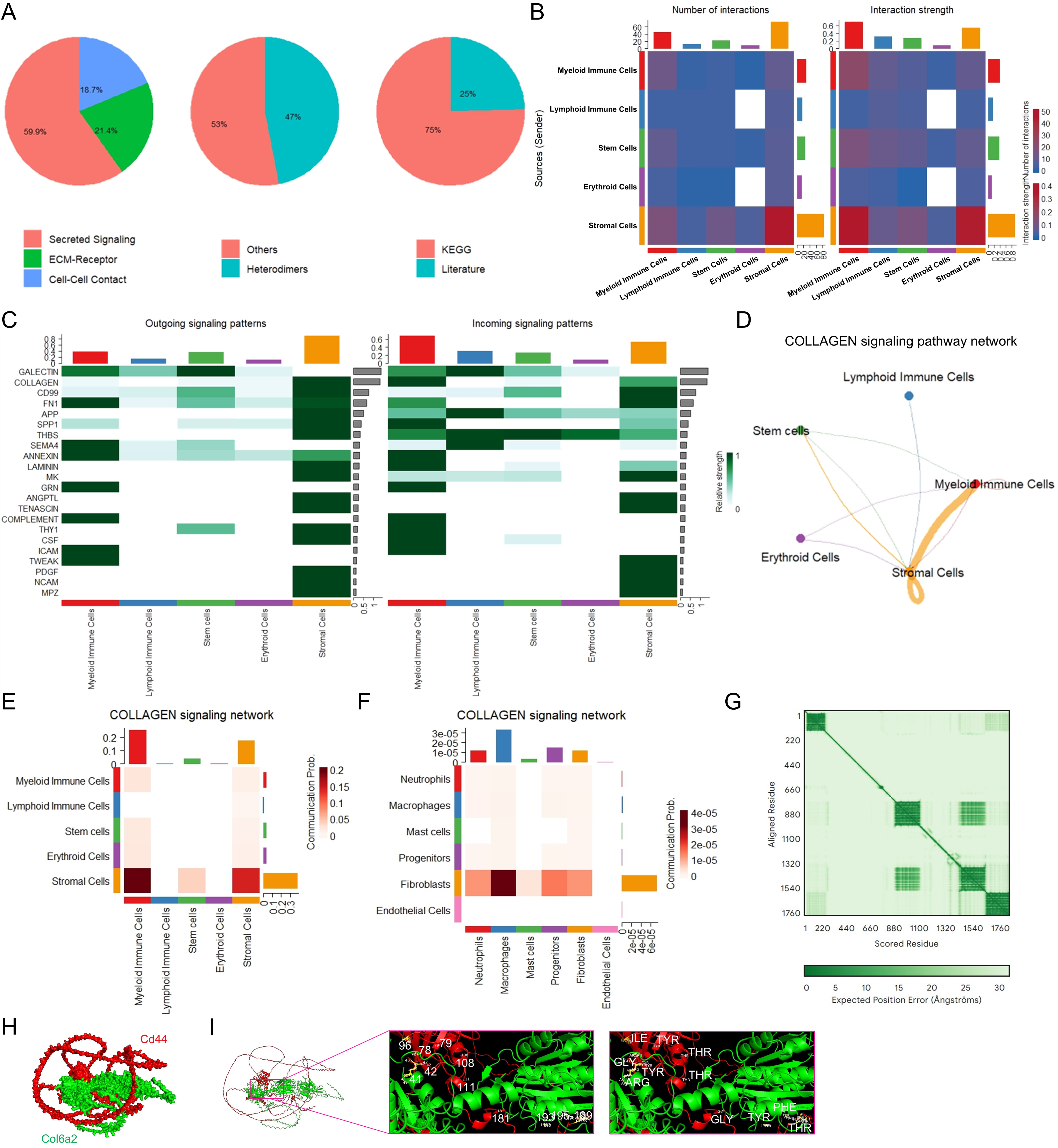


**Fig. S4** Intercellular crosstalk and ligand-receptor pair prediction in cells surrounding ZrO_2_ implants. (A) Proportions of autocrine/paracrine signaling interactions, extracellular matrix (ECM)-receptor interactions, and cell-cell contact interactions in the CellChat interaction database. (B) Heatmap visualization of intercellular interaction counts and strengths. (C) Visualization of Outgoing-Incoming signaling patterns and interaction strengths. (D) COLLAGEN signaling pathway network. (E, F) Outgoing-Incoming network of COLLAGEN signaling in total cells and cell subpopulations. (G) AlphaFold3-based structure prediction of COL6A2 (heatmap representation). (H, I) Molecular dynamics simulation and binding site prediction of COL6A2 and CD44.


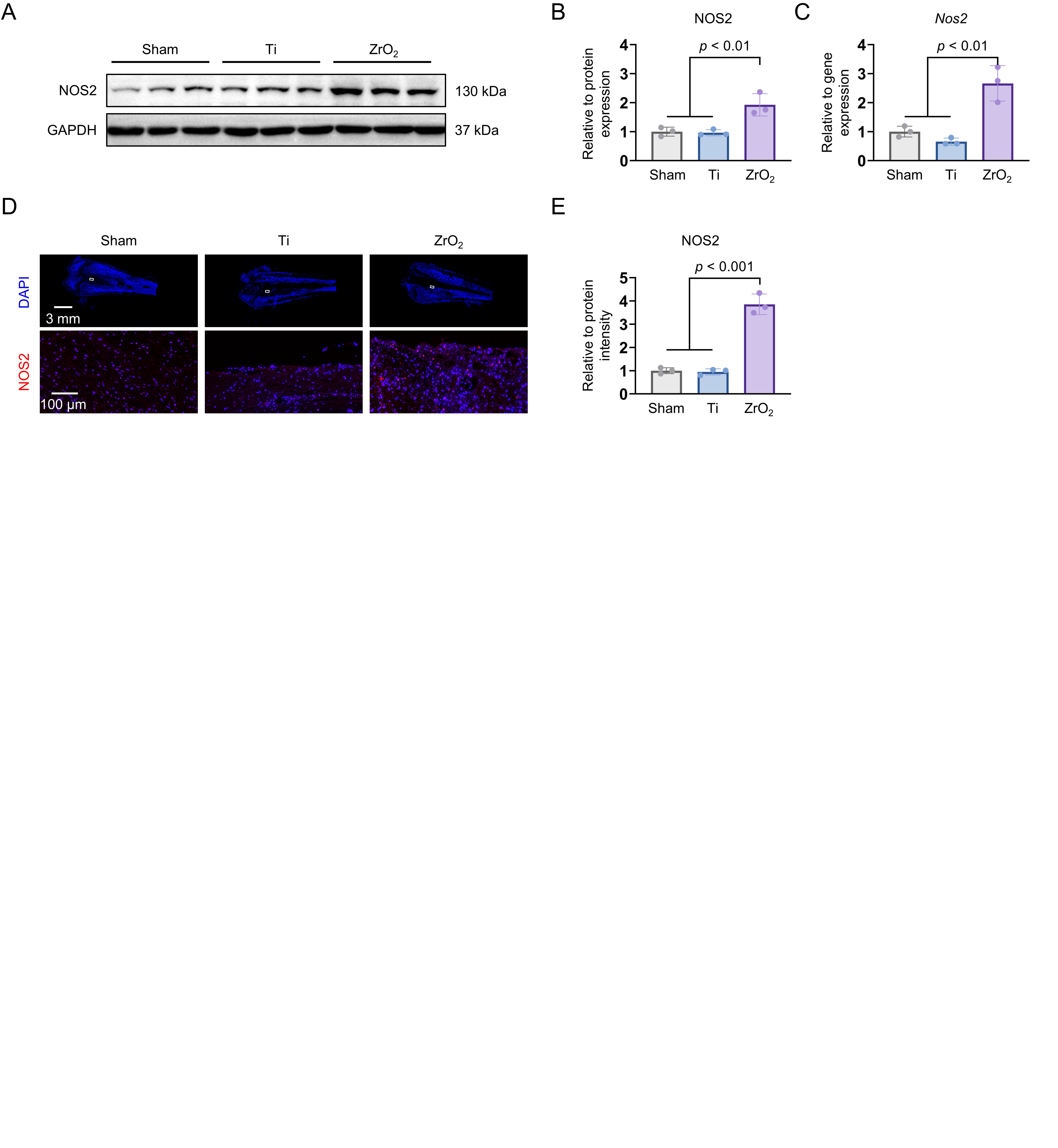


**Fig. S5** ZrO₂ implantation induces inflammatory macrophage activation. (A) Expression analysis of the inflammatory macrophage marker protein NOS2 in peri-implant bone marrow tissue. (B) Statistical analysis of NOS2 protein expression. (C) Transcriptional level quantification of the inflammatory macrophage marker gene *Nos2*. (D) Immunofluorescence localization of inflammatory macrophages in peri-implant bone marrow tissue. Scale bars: 3 mm and 100 μm. (E) Statistical analysis of inflammatory macrophage fluorescence intensity. Statistical significance was determined using one-way ANOVA.


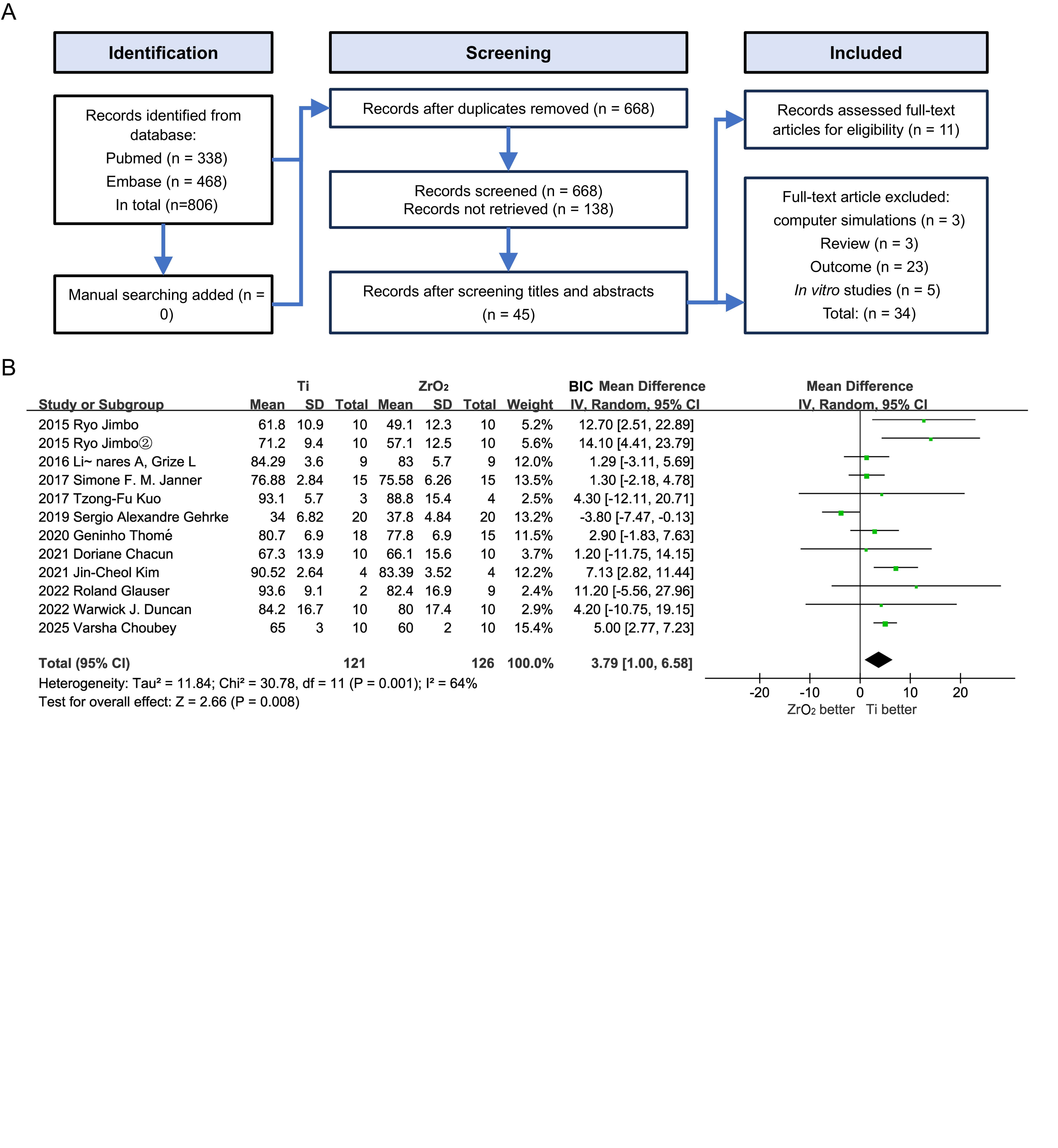


**Fig. S6** Meta-analysis of bone-implant contact (BIC) for Ti and ZrO₂ implants. (A) Flowchart of the meta-analysis. (B) Forest plot of the meta-analysis. Statistical significance of the pooled effect was assessed using an inverse-variance weighting model.


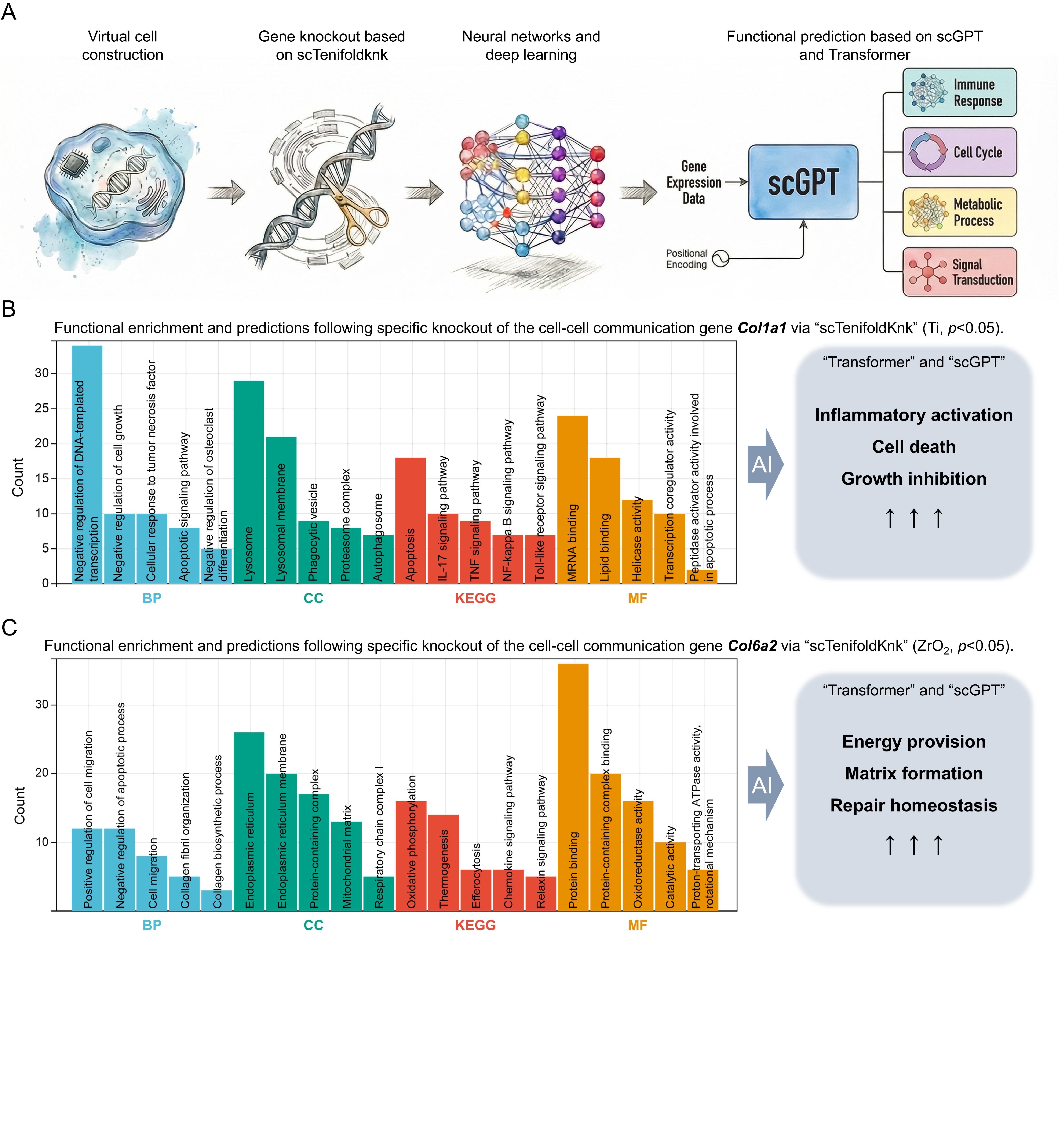


**Fig. S7** Virtual gene knockout and functional analysis. (A) Flow chart of virtual knockout-based analysis (Image generation based on “Diffusion Models” and “Flow Matching”). (B) Cell-cell communication gene *Col1a1* was specifically knocked out in the Ti group using “scTenifoldKnk”, and its function was subsequently predicted via “scGPT” and “Transformer” models. (C) Cell-cell communication gene *Col6a2* was specifically knocked out in the ZrO_2_ group using “scTenifoldKnk”, and its function was subsequently predicted via “scGPT” and “Transformer” models.
